# Supplementary material for: Reconstruction of macroglia and adult neurogenesis evolution through cross-species single-cell transcriptomic analyses
Source: Nat Commun. 2024 Apr 17;15:3306. doi: 10.1038/s41467-024-47484-1 (PMC11024210; doi:10.1038/s41467-024-47484-1)
Supplement: Supplementary file 1 — Supplementary information [file 41467_2024_47484_MOESM1_ESM.pdf]

Supplementary Information for

Reconstruction of macroglia and adult neurogenesis evolution through cross-species single-cell transcriptomic analyses

David Morizet, Isabelle Foucher, Alessandro Alunni, Laure Bally-Cuif

Corresponding authors: david.morizet@pasteur.fr; laure.bally-cuif@pasteur.fr

This file includes:

Supplementary text	p.1-6
Figs. S1 to S20 and legends	p.7-27
Supplementary Table 1	p.28-29
Supplementary References	p.29-34

Supplementary text

Homology of neurogenic niches

Regionalization of the SVZ niche in the mouse brain has been the object of study for many years [1–10]. The mouse SVZ can be subdivided into several microdomains with distinct molecular signatures, correlated to the type of progeny the stem cells can produce and to the developmental domain they originate from. In particular, distinct domains of the pallium and subpallium give rise to specific regions along the dorsoventral axis of the adult neurogenic niche, with neural stem cells retaining expression of transcription factors from the developmental period to adulthood. Adult neurogenesis in the zebrafish telencephalon has been almost exclusively studied in the dorsomedial palium (Dm region) [11]. Although anatomical subdivisions of the telencephalon have been proposed based on the layout of neurons [11], no direct relationships linking the different ventricular territories between the everted telencephalon of actinopterygians and the evaginated telencephalon of most other vertebrates had been established.

Our work shows that major dorsoventral subdivisions along the telencephalic ventricle in zebrafish mirror those found in mammals, and in particular that Dm is likely homologous to the dorsal wall of the lateral ventricle which harbors deeply quiescent neural stem cells in the mouse [8,10]. This corrects a previously proposed territorial subdivision of the telencephalic ventricles [12] and lays the groundwork for further comparisons across

vertebrates. For example, RG in Dm proliferate less than RG from the *gsx2* domain in the adult zebrafish telencephalon [13]. This mirrors the relative levels of proliferation between the dorsal and lateral walls of the ventricles in adult mouse and suggests that functional correlates of positional identity are also conserved to some extent. In addition, although it has been shown that a structure reminiscent of the rostral migratory stream exists in the zebrafish telencephalon [14], whether the neurons that are generated are similar to the newborn olfactory neurons in mammals and whether different regions of the ventricle contribute in the same way is unknown. It is believed that the olfactory systems from mammals and zebrafish share many similarities including a homologous pallial area with sophisticated signal-processing[15]. Yet, mammals are believed to be more reliant on olfaction and to have benefited from adaptations linked to breathing which allow them to inhale through the nose whereas olfaction relies on passive collection of odorants in fish. Further studies, combining molecular phenotyping, functional assays and lineage tracing in zebrafish while leveraging knowledge from the results obtained in mammals, could shed light on the contribution of adult neurogenesis to olfaction in zebrafish, how it compares to the same process in mammals and highlight potential adaptations related to adult neurogenesis that happened over the course of evolution to improve olfaction.

We did not find support in favor of the existence of a population homologous to dentate gyrus granule cells in our data. This could be due in part to a limitation of our approach which aimed at enriching for glia and thus likely underrepresents the full diversity of neuronal populations in the zebrafish telencephalon. Although the extra-ventricular position of the hippocampus appears to be mammalian-specific, recent data confirms that the different hippocampal subfields are also individualized in reptiles [16] . However, in urodele amphibians this appears to not be the case [17]and instead neurons co-expressing CA and dentate gyrus markers are found in the medial pallium [18]. Nonetheless, our results and previous studies [19] show that gradients of expression involved in specifying the hippocampal pallium are present and functional investigations have identified a region of the caudal dorsolateral zebrafish pallium that is involved in spatial encoding [20,21], a key function ascribed to the hippocampus in mammals. Thus, it is uncertain how closely stem cells from the hippocampal pallium might be related between species and to what extent this might be correlated with cell type diversification in the mammalian medial pallium. We could not identify a subpopulation of cells from the *wnt3a+* domain previously observed with in situ hybridizations [19], likely because these cells are very few in number and might also have been selected against during our dissection. A medio-lateral gradient of expression of morphogens in RG was described in axolotl with molecular signatures reminiscent of those in mammals and reliably delineating different regions of the pallium[17]. Similarly, in our re-analysis of *Pogona vitticeps* data [22], we found that a subset of RG expressed high levels of *WNT3A* which displays medial pallium expression in mammals. Unfortunately, neither *WNT3A* nor *PROX1* were reliably detected in a pioneering spatial transcriptomic approach on *Pogona vitticeps* telencephalon [23], which prevented us from confirming that the *WNT3A*-expressing RG are in the same territory as the dentate gyrus-like neurons.

We anticipate that these remaining questions will be addressed soon through larger datasets on teleost brains and targeted studies across vertebrates which will shed light on the origin of cell type diversification in the hippocampus. This is of particular interest for the study of adult neurogenesis as the dentate gyrus niche has been described as being more of a mammalian innovation [24] whereas the SVZ would be more of a vestigial trait, despite the fact that the architecture of the mammalian SVZ is also noticeably different from the ventricular zones of non-mammalian vertebrates. The similarities in RG regional patterning and functional regulation of neurogenesis activity across evolution [25] as well as the architectural divergences between both mammalian telencephalic niches and homologous regions in non-mammalian species argue against this hypothesis, but

a greater understanding in the molecular and lineage structure similarities across vertebrate will be necessary to substantiate or definitively invalidate it.

A mechanistic hypothesis on the rarefaction of adult neural stem cells in humans

The existence of adult neurogenesis in humans has been a highly debated topic since it was first hypothesized. In the last few years several studies investigating human neurogenesis have reported contradicting results and reignited the debate [26–33]. Single-cell RNAseq datasets from the human and non-human primate hippocampus have been generated with some claiming to detect neural stem cells. We reanalyzed these datasets [34–38] but we were unable to reconstruct a complete neurogenic cascade in humans or macaque. In particular our reanalysis is consistent with another report [31] that concluded that cells initially described as neural stem cells [37] were in fact likely ependymocytes. Although there is some evidence that immature neurons can still be detected in adult humans [29,32] recent studies have shown that some neurons can retain both molecular and functional features of immature neurons a long time after being generated [39–41]. Such neurons have been termed non-newly born immature neurons, or dormant precursors, and their existence makes it difficult to confirm that other neurons expressing immature neuron markers are indeed the product of ongoing adult neurogenesis. Moreover, adult neural stem cells at the root of neurogenesis remain elusive and it is more largely agreed that they are likely prematurely depleted compared to most other species [42]. However, we lack a conceptual framework to explain how and why such a situation might have arisen despite the advantages of ongoing adult neurogenesis with respect to functional plasticity and regenerative abilities. Our analysis of the molecular cascade involved in neurogenesis regulation highlighted the important role of the Notch pathway. Strikingly, the main Notch receptor expressed in quiescent RG is different in mammals compared to lizards, amphibians and fish, suggesting that a switch between paralog resulted in *NOTCH2* taking up the functions of *NOTCH3* in mammals. Such switches between paralogs are common during evolution[43]. It was recently shown that human-specific genes derived from duplication of the *NOTCH2* locus –the *NOTCH2NL* genes– potentiate regular Notch signaling and promote symmetric divisions in RG early on, which ultimately results in an enlarged cortex [44,45]. Other studies also showed that potentiating Notch signaling could result in longer maintenance of RG early on, but that this is later followed by an increased terminal differentiation into astrocytes [46]. Taking all this into account, the switch between *Notch3* and *Notch2* in mammals, leading to durable co-expression of *NOTCH2* and *NOTCH2NL* genes in human RG [44,47], could lead to accelerated RG depletion and the lack of RG-like cells in adult humans. This model would explain why this change is specific of humans while other non-human primates, which do not have functional *NOTCH2NL* genes, retain adult neurogenesis, as has been demonstrated by radiographic, histologic and sequencing studies [48–50,36]. Some arguments explain the low neurogenesis in humans based on a selection against plasticity due to the size and complexity of their brain[51]. Our interpretation suggests a tradeoff with an expanded neocortex linked to developmental processes rather than a necessity to repress adult neurogenesis. Selection for these traits would have taken place at a moment when sight was already the primary sensory modality thus reducing selective pressure on olfactory processing [52], and when life expectancy was much shorter, thus resulting in only a short period of time without hippocampal neurogenesis given that it persists well into teenage years. Interestingly, previous allometric studies have identified a positive correlation between the sizes of the hippocampus and of olfactory structures in mammals and an inverse relationship between the size of these regions and that of the isocortex[53]. This inverse relationship is particularly noticeable in species that rely heavily on sight rather than olfaction such as simians [54], which is consistent with the idea that selective

pressure to maintain adult neurogenesis in early humans might have been relaxed. Most importantly, this model can be experimentally tested. First, several experiments modulating the Notch pathway have been conducted with results supporting the proposed hypothesis [46,55–60]. Second, copy number variations in the *NOTCH2NL* locus are a well-known cause of neurodevelopmental disorders leading to the creation of patient cohorts [44,47]. Using current work aimed at developing ways of quantifying the presence of stem cells in vivo [30,61], or post-mortem histological studies on tissue from patients with fewer *NOTCH2NL* copies, will make it possible to eventually found further correlates in support of our model directly in humans.

Emergence and diversification of glia

Relatively little is known about the evolutionary origins of glia [62]. Several phyla appear to contain representatives both with, or apparently devoid of, glial cells. Moreover, glial cells are much more diverse than neurons in appearance, molecular profile and functions. Whether glia emerged or not alongside neurons is disputed and what they emerged from is unknown. It has been proposed that, because neurons are very demanding energetically and inefficient at controlling the extracellular changes caused by their activity, glial cells taking up these functions must have emerged at the same time as neurons in epithelial nerve nets [63]. However, not only do some species appear to not have any glial cells, but in some species with glial cells, like *C. elegans*, these are not necessary for survival [64,65]. Moreover, neurons can perform functions often associated with glial cells, for example in copepod where conduction speed is increased by a sheath derived from neurons themselves [66]. Our comparative analysis also revealed cases (e.g., in Ciona, Fig.S16) where genes usually associated with glial functions appear expressed at high levels in neurons instead. We do not believe that our approach is powerful enough to confirm that neurons in species with a limited glia complement are less dependent on support cells. However, in light of descriptions from the literature and our observations, we do believe that functionally investigating this hypothesis is worthwhile, for example by assessing Cnidarian neurons' abilities to recapture neurotransmitters and provide their own source of energy. It is apparent that neurons and glia have not evolved separately but rather have co-evolved, and it would be interesting to determine whether this has resulted in neurons becoming less self-sufficient in presence of glia.

The astroglial system of actinopterygii has been described as having undergone only moderate evolutionary modifications, and they are generally thought to lack bona fide astrocytes [67]. This implied that astrocytic functions were either unnecessary or fulfilled by another cell type, with RG as the most likely candidate to do so [68]. Although recent work showed that hindbrain RG in zebrafish were capable of acquiring intricate morphologies [69] and communicating with neurons [70], these abilities are shared between astrocytes and RG-like cells in mammals [71–78]. Thus, while these results highlight fundamental properties of RG, they cannot by themselves confirm the hypothesis that zebrafish RG can behave like astrocytes.

Here, we found that distinctions among RG were partially driven by a group of genes which also separates astrocytes from RG-like cells in mammals. Within the limits of scRNAseq and quantifications using a few markers with RNAScope, we almost never observe proliferating or *ascl1a*+ cells that express high levels of astrocytic markers, although clonal analysis suggests that all RG in the zebrafish pallium eventually give rise to neurons. This suggests that the majority of astrocytic-like cells recovered in the scRNAseq data correspond to a substate of quiescence for RG. This implies that their ability to fully perform support functions is coupled to state transitions: astrocytic genes are expressed at high levels when the cells are deeply quiescent and turned off as they activate. This suggests that before subfunctionalization and the emergence of distinct cell types fulfilling those roles in terrestrial vertebrates, ancestral astroglial cells underwent a form of temporal cell differentiation allowing them

to perform both functions but not simultaneously. This is reminiscent of the current model of metazoan evolution based on observations of unicellular organisms capable of colony formation [79,80]. This sequential dimension differs from previous examples of cell-type evolution described in the brain with ancestral neurons that simultaneously co-expressed sets of genes that became segregated after subfunctionalization [16]. This also has important implications for our understanding of quiescence and differentiation. It shows that contrary to a popular representation the quiescence state is not —or at least not necessarily— a state of rest but can rather be an opportunity for a stem cell to fulfill functions not related to the production of progeny. Moreover, if cells physiologically oscillate between a state where they are ready to activate and produce progeny and a state in which they are quiescent and performing functions usually associated with differentiated cells, we must be very careful in defining cases of regenerative de-differentiation.

Our approach does not allow us to pinpoint the time when cells emerged that display permanent astrocytic functions and no physiological ability to generate neurons. Such cells could have first appeared without losing their contacts with the ventricles and be present in some of the data we have analyzed. However, without a reliable quantification of the proportion of RG capable of generating neurons, we cannot support or invalidate this possibility. Delamination of astroglia to give rise to parenchymal astrocytes appears to be linked to parenchymal thickness. It has been hypothesized that brain enlargement leads to stretching of the radial process which itself causes delamination [81]. Work from the Kalmán lab, in particular on crocodilians, suggests that the number of parenchymal astrocytes in species with low numbers of them is not directly correlated to thickness [82]. However, close crocodilian relatives with thicker brains show that the enlarged areas are areas that likely contained comparatively higher numbers of astrocytes in archosaurian ancestors. This suggests that parenchymal astrocytes can appear without a substantial thickening of the parenchyma and then engage in a self-reinforcing loop. On one hand the presence of dedicated parenchymal astrocytes which can more efficiently fulfill support functions might be beneficial to cope with increased metabolic needs [83] and relax constraints on parenchymal thickness. On the other hand, the increase in thickness itself can promote more astroglial delamination to give rise to parenchymal astrocytes.

Independently of whether parenchymal astrocytes precede or follow parenchymal enlargement, a large parenchyma is associated with large numbers of parenchymal astrocytes and with reduced RG numbers. Birds are remarkable in this regard, as they have very thick brain parenchyma and a large number of astrocytes, yet also retain large numbers of RG acting as neural stem cells comparatively to mammals, squalomorphs [84] or hagfish [85]. It would thus be interesting to turn back to birds, which played a major role in the early days of the study of adult neurogenesis [86,87], to better understand how the balance between astrocytic differentiation and self-renewal of neural stem cells can be regulated. Further comparative studies across species harboring parenchymal astroglia will also allow us to determine the level of similarity between these cells and their generative process. This would in turn shed light on whether the differentiation landscape of RG is limited. Hagfish and platypus represent two highly interesting unconventional models to characterize in this context. Recent work on lamprey suggests that their RG co-express astroglial and oligodendroglial genes [88] and that they exhibit morphological traits consistent with a dual function [89]. Assuming that this is the ancestral vertebrate state, the nature and behavior of hagfish parenchymal glia are highly intriguing, and they might represent yet another sister cell type to other glia with features of both astrocytes and oligodendrocytes. Similarly, one study reported that contrary to other mammals, monotremes appear to not have distinct oligodendrocytes and astroglial cells but rather a hybrid cell type [90] (although these results have never been corroborated to our knowledge). Given efforts to improve the usefulness of hagfish [91,92] as a model and with single-cell transcriptomics having already been applied

to platypus [93] it is likely that in the near future studies will answer these questions and further improve the resolution of the phylogenetic tree of glial cell types.

Regarding glial evolution at a larger scale, and more specifically homology of glial cells among Nephrozoa, the conclusions we draw differ from a recent report[94]. In this study the authors integrated single-cell RNAseq from fly, octopus and mouse brains and showed that ensheathing glia in flies and some glial cells in octopus clustered together with telencephalic astrocytes. They identified a restricted set of genes expressed in these cells across species and used this to propose that glial cells across bilaterians are homologous. Our approach differs from theirs in that we first aimed to identify sets of genes to reliably characterize vertebrate glia, in particular astroglia, and then mapped those signatures onto data derived from many species. Using this method, we did not find any cell type in invertebrates that on its own exhibits a transcriptional profile similar enough to a type of vertebrate glial cells to be confidently identified as homologous. That is not to say that we did not find similarities. Consistently with Styfhals et al. [94], we found glial cells expressing an ortholog of a Notch receptor, a transporter responsible for GABA uptake, a transporter responsible for glutamate uptake, and glutamine synthetase which is responsible for producing glutamine from captured glutamate that in turns serves as a substrate for neurons to produce more glutamate and/or GABA not only in representatives of vertebrates, insects and mollusks but also of plathelminths. However, orthologs of transcription factors that are known to be essential to define the identity of vertebrate glial cells such as *SOX9*, *SOX2*, *SOX10*, *NKX2.2* or *OLIG2* were not expressed in invertebrate glial cells even when they were abundantly detected in a dataset. Conversely, the “glial cells missing” gene which is essential for glia generation in insects[95] does not exist in any deuterostome genome. Thus, overall, we found that only a relatively small fraction of informative genes were co-expressed in invertebrate glia and that there was little evidence for a shared coregulatory complex. The only example of a major transcription factor involved in glial specification that was found across bilaterians was the presence of a Notch receptor ortholog. Notch receptors are expressed in many cell types and a single transcription factor being shared cannot by itself point towards a common ancestral regulatory complex. In absence of shared regulators despite the shared expression of a few effector genes and taking into account accumulated evidence about the ontogenetic differences between glial cells in insects and vertebrates, we therefore propose that similarities across bilaterian glia arose in large part through homoplasy.

However, we do not rule out that to some extent the transcriptional profiles of glial cells across bilaterians reflects remnants from a state that preceded glial cells as we currently know them. In particular, the genes that Styfhals et al. [94] and ourselves identify as being shared in several phyla might indeed have already been expressed in an ancestral cell type, and maintained possibly because of their importance in generating a functional nervous system.

Supplementary Figures

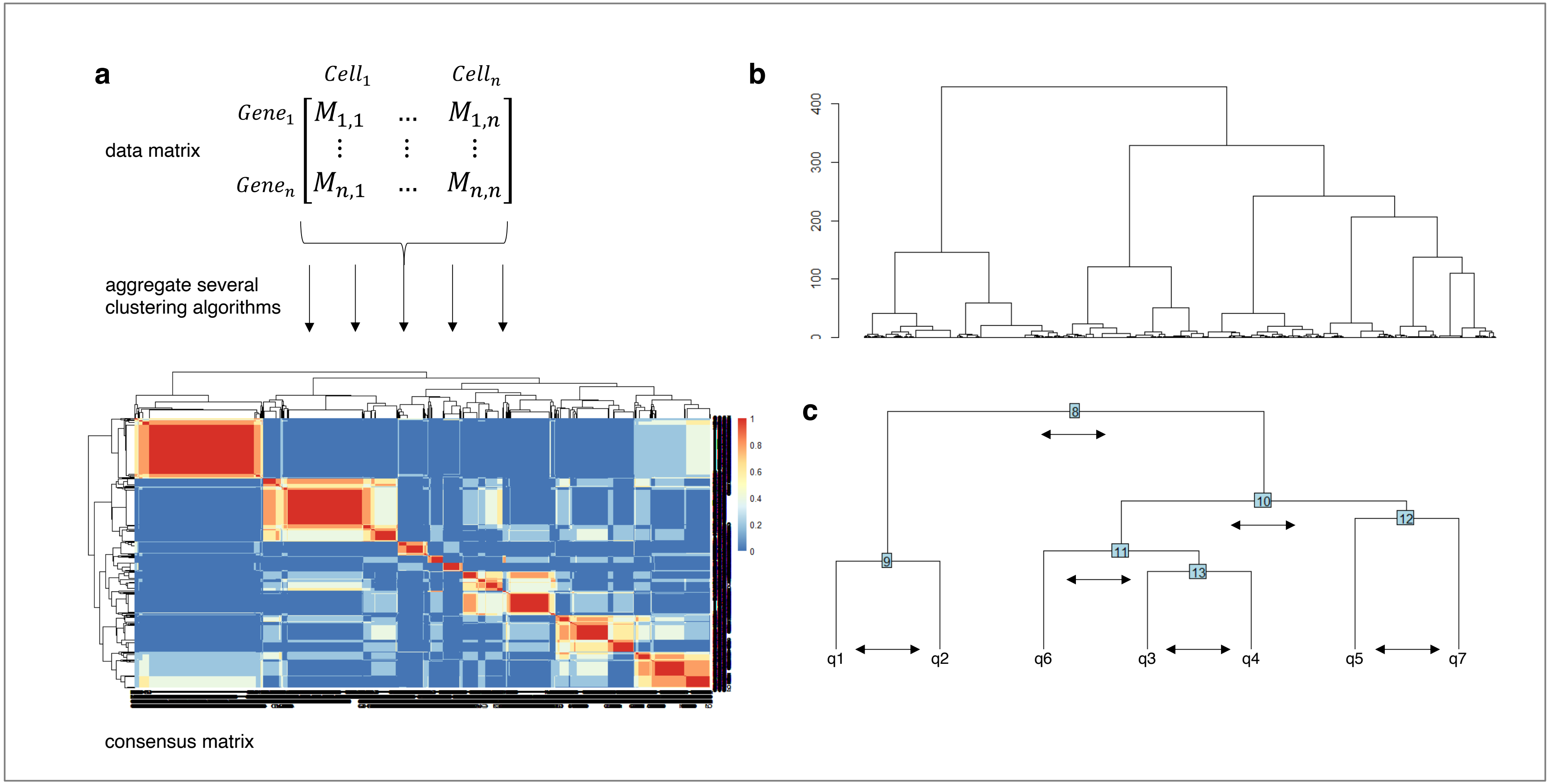

Supplementary data Fig.1. Approach used for clustering quiescent radial glia. a, Schematic of the consensus clustering approach. **b,** Dendrogram obtained from hierarchical clustering on the final consensus matrix for the entire dataset. **c,** Dendrogram of final qNSC clusters, arrows depict the combinations that would be subjected to differential gene expression analysis to determine whether they need to be merged or not.

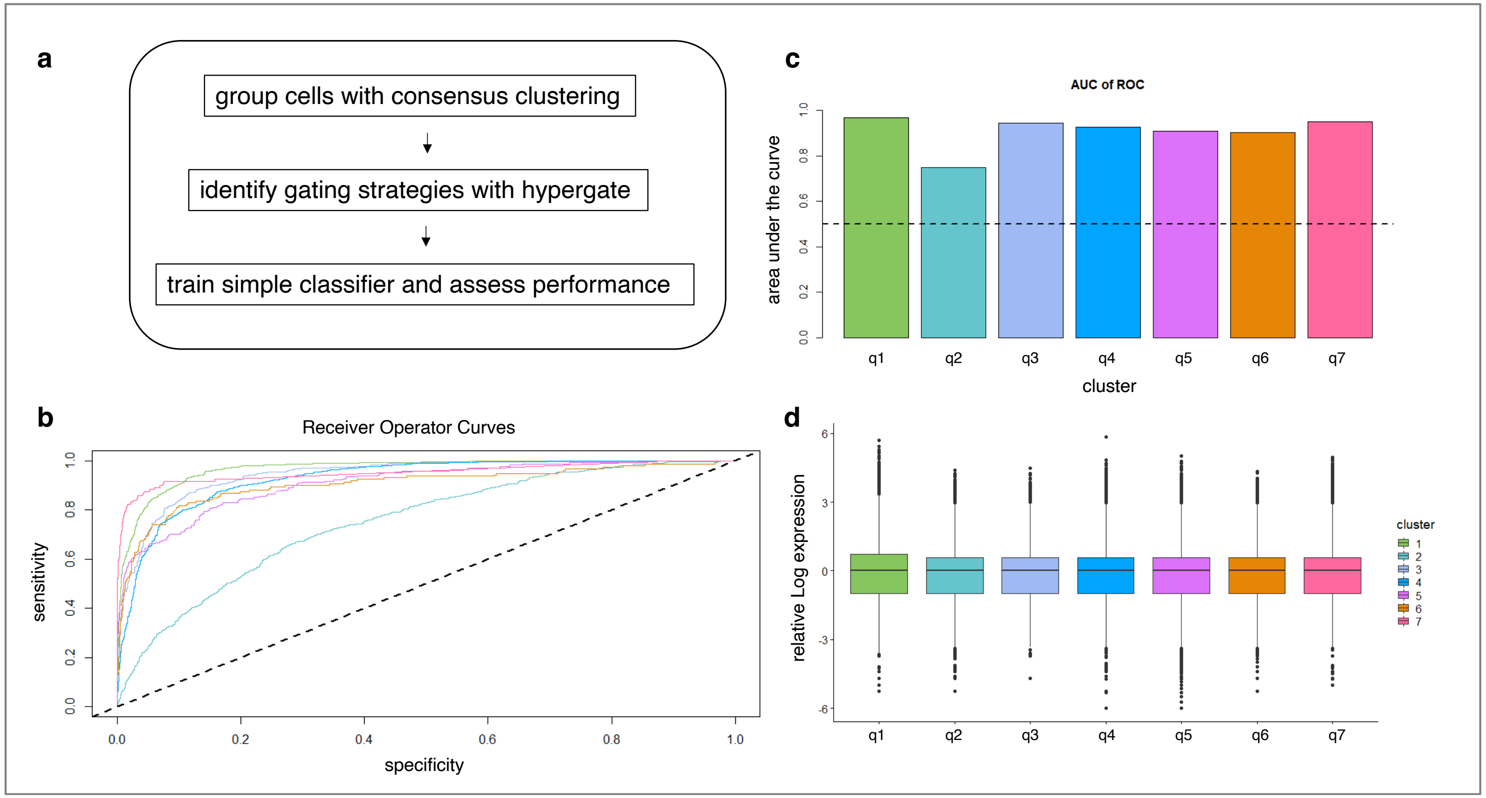

Supplementary data Fig.2. Verification of the validity of clustering. **a,** Schematic of the approach used to verify cluster actionability, inspired by Valentine Svensson (https://www.nxn.se/valent/2018/3/5/actionable-scrna-seq-clusters). **b,** Receiver Operator Curves depicting how well clusters are recovered with the unfiltered output from hypergate, dashed line represents performance expected by chance, line color matches cluster colors used in other figures. **c,** Area under the Receiver Operator Curves from B, dashed line represents performance expected by chance. All clusters get an AUC over 0.7. q2 likely being in an intermediate stage between q4, q3 and q1, was expected to get the lowest performance. **d,** Relative Log Expression for all genes across all clusters. The distributions are centered around 0 in all cases suggesting that clustering is not driven by cell quality.

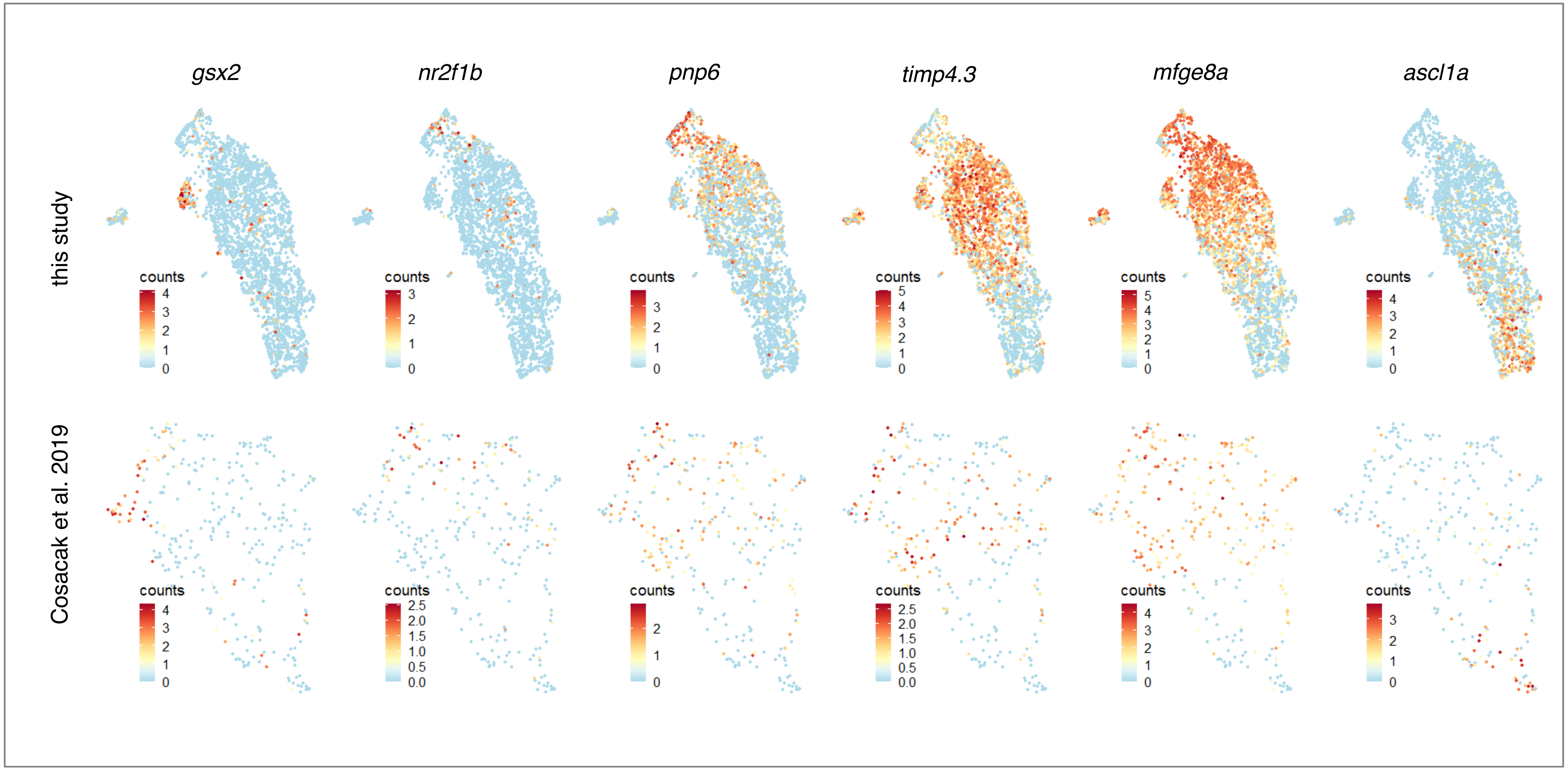

Supplementary data Fig.3. UMAP expression of cluster markers in qRG. Expression of genes enriched in different clusters in our dataset and comparison with a previously published dataset that profiled 10 times fewer cells [12], here reanalyzed with our clustering method (Supplementary data Fig.S1). Gene expression patterns show the same relative distribution in both datasets (coexpressed genes in one dataset are coexpressed in the other, genes restricted to a small subset of cells are equally restricted in both datasets…) and consistent with our proposed grouping of qRGs rather than the one proposed in [12].

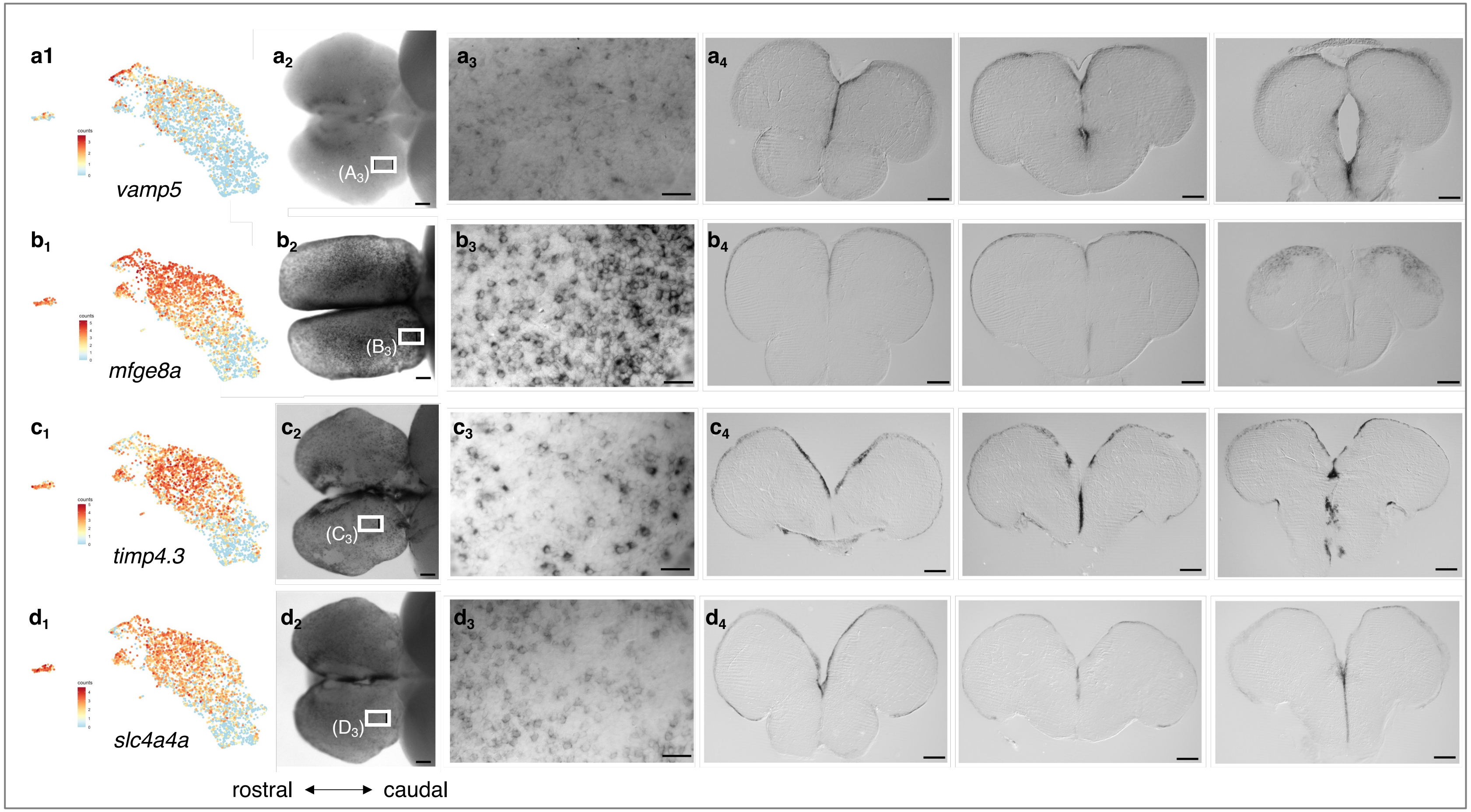

Supplementary data Fig.4. In situ validation of genes that show variable expression among qRG in the zebrafish adult pallium. Chromogenic ISH validates the relative extent of expression of genes enriched in subsets of qRGs and confirms the existence of distinct qRG populations intermingled with each other in the zebrafish adult pallium. Each line follows the same pattern with gene expression plotted on the qRG UMAP (**a_1,b_1,c_1,d_1**), a whole mount image in dorsal view (**a_2,b_2,c_2,d_2**), a close-up of a region in the caudal part of the dorsal pallium (boxed on the whole mount image) (**a_3,b_3,c_3,d_3**), and three coronal slices at different positions along the rostro-caudal axis from rostral to caudal (**a_4,b_4,c_4,d_4**). Scale bars≈500µm.

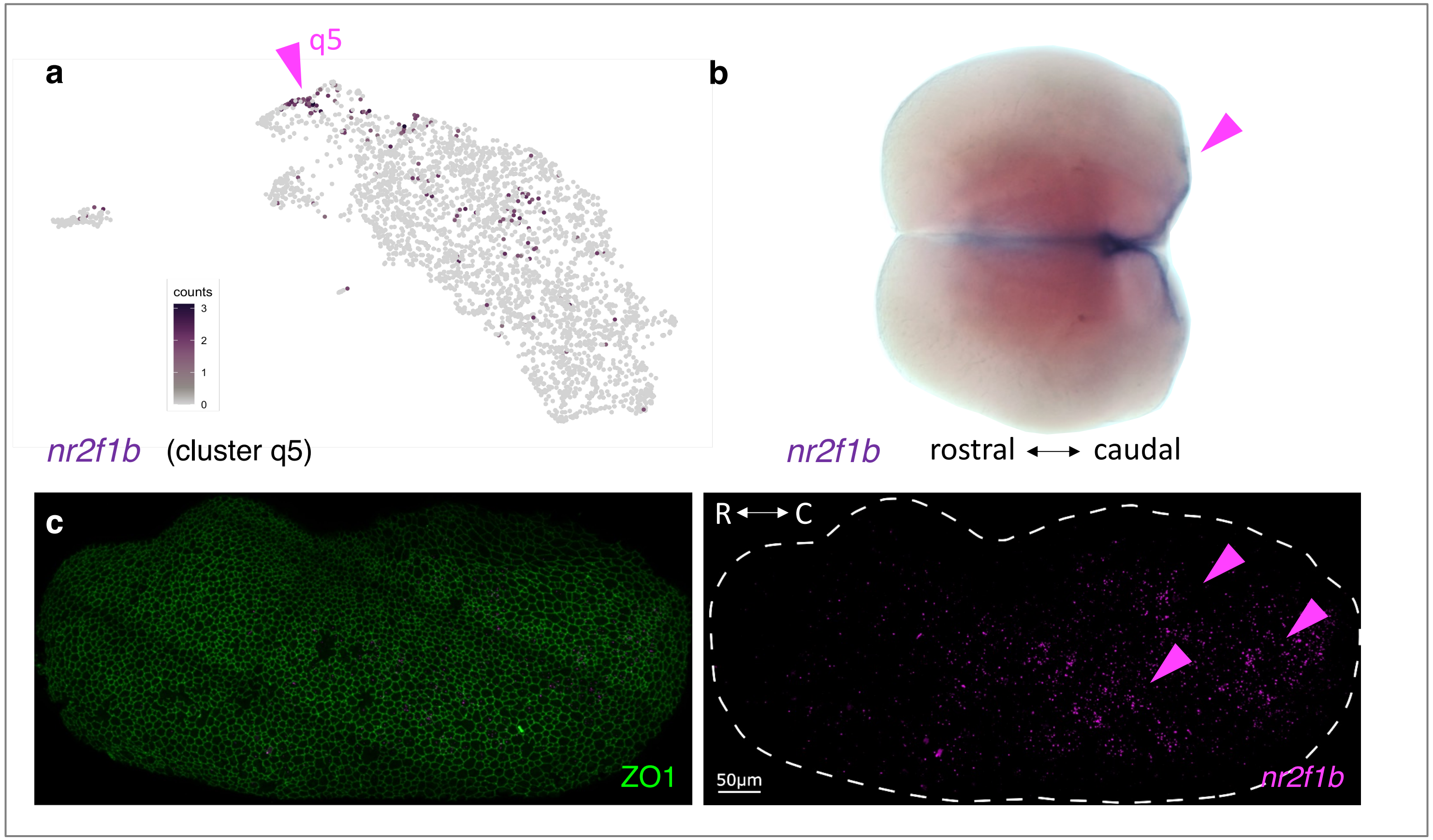

Supplementary data Fig.5. Caudo-rostral gradient of *nr2f1b* expression in the zebrafish adult pallium. **a,** *nr2f1b* expression projected on the qRG UMAP, showing specificity of expression in q5 (arrowhead, see also Fig.2B). **b,** Dorsal view of a zebrafish telencephalon stained for *nr2f1b* expression by chromogenic ISH. **c,** Dorsal view of a hemisphere of a zebrafish telencephalon (confocal microscopy, z=20µm projection), double stained in whole-mount for ZO1 (green, immunohistochemistry) and *nr2f1b* expression (magenta, RNAScope ISH). The two channels are shown separately to appreciate the caudo-rostral gradient of *nr2f1b* expression.

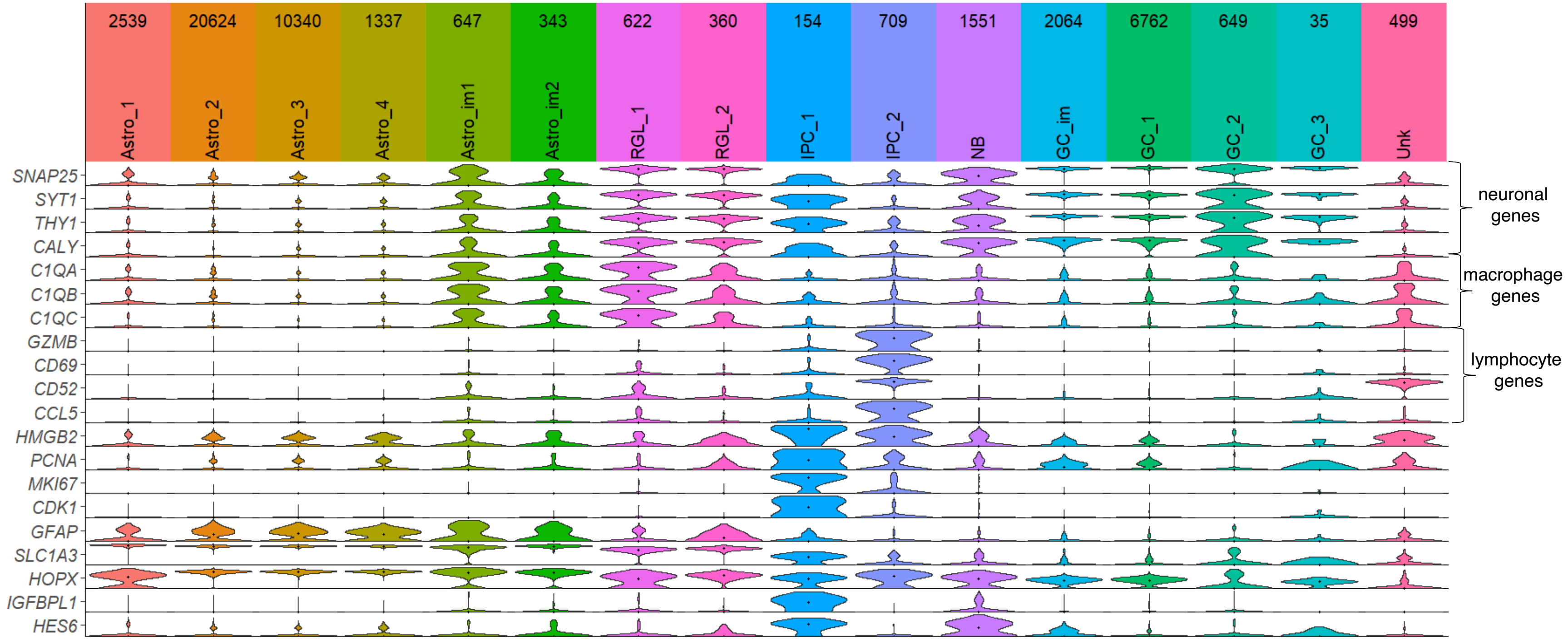

Supplementary data Fig.6. Re-analysis of Macaque dentate gyrus scRNAseq [36]. Violin Plot for selected genes in an adult macaque hippocampus dataset with the initially proposed cluster identities (re-clustering gave comparable results). We included astrocytic clusters as well as clusters proposed to be involved in adult neurogenesis. *HOPX* is one of the genes most enriched in RG-like cells (RGL) over astrocytes in mice but is expressed at lower levels in putative macaque RGL clusters which also express several genes supposedly specific of neurons or macrophages suggesting that those are multiplets. Putative IPC_2 expresses several lymphocyte-specific genes and is likely a population of cycling blood cells. IPC_1 expresses intermediate levels of astrocytic and neuronal genes and high expression of cell-cycle related genes, consistent with its annotation. *IGFBPL1* and *HES6* show high expression in IPC_1 and negligible expression in clusters other than IPC_1 and neuroblasts, making them valuable candidates to distinguish between continued neurogenesis or persistence of DCX in developmentally-born neurons in humans.
Astro: Astrocytes, GC: granule cells, IPC: intermediate progenitor cells, NB: neuroblasts, RGL: radial glia-like cells, Unk: unknown. Im: immature.

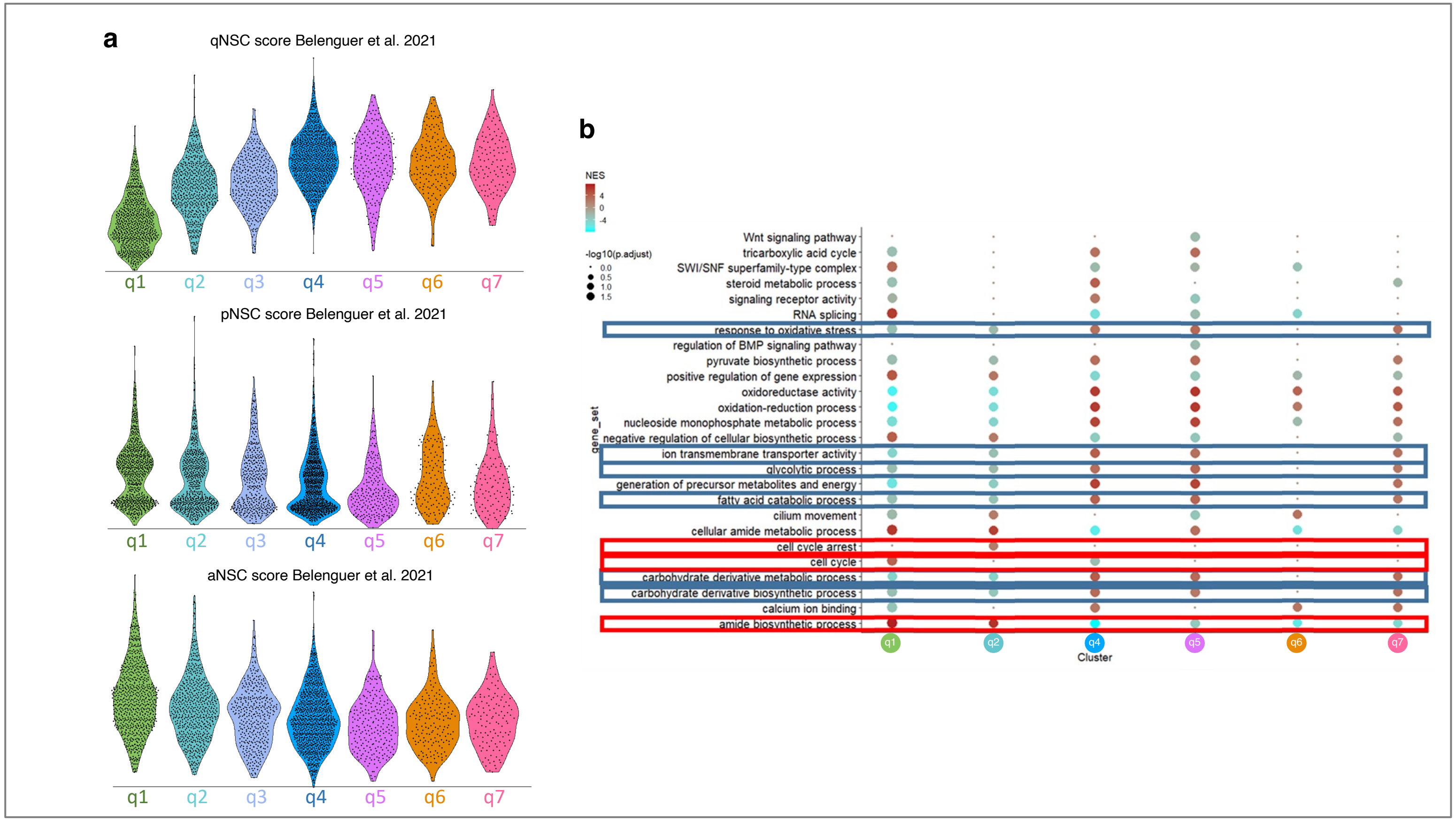

Supplementary data Fig.7. qRG are in different depths of quiescence. **a,** Violin plot depicting, in zebrafish telencephalic qRG clusters, the expression levels of genes the orthologs of which are transcriptionally associated with quiescent, primed and activated RG (respectively labeled qNSC, pSNC and aNSC) in mice (dataset derived from [96]). Note the graded representation of q1 to q4 signatures along the a→p→q progression. **b,** Gene Set Enrichment Analysis on zebrafish qRG clusters. Gene sets highlighted in blue and red are associated with quiescence and activation, respectively. One column for each cluster, except for q3, which was too close to q4 to identify specific gene sets and is not depicted individually here.

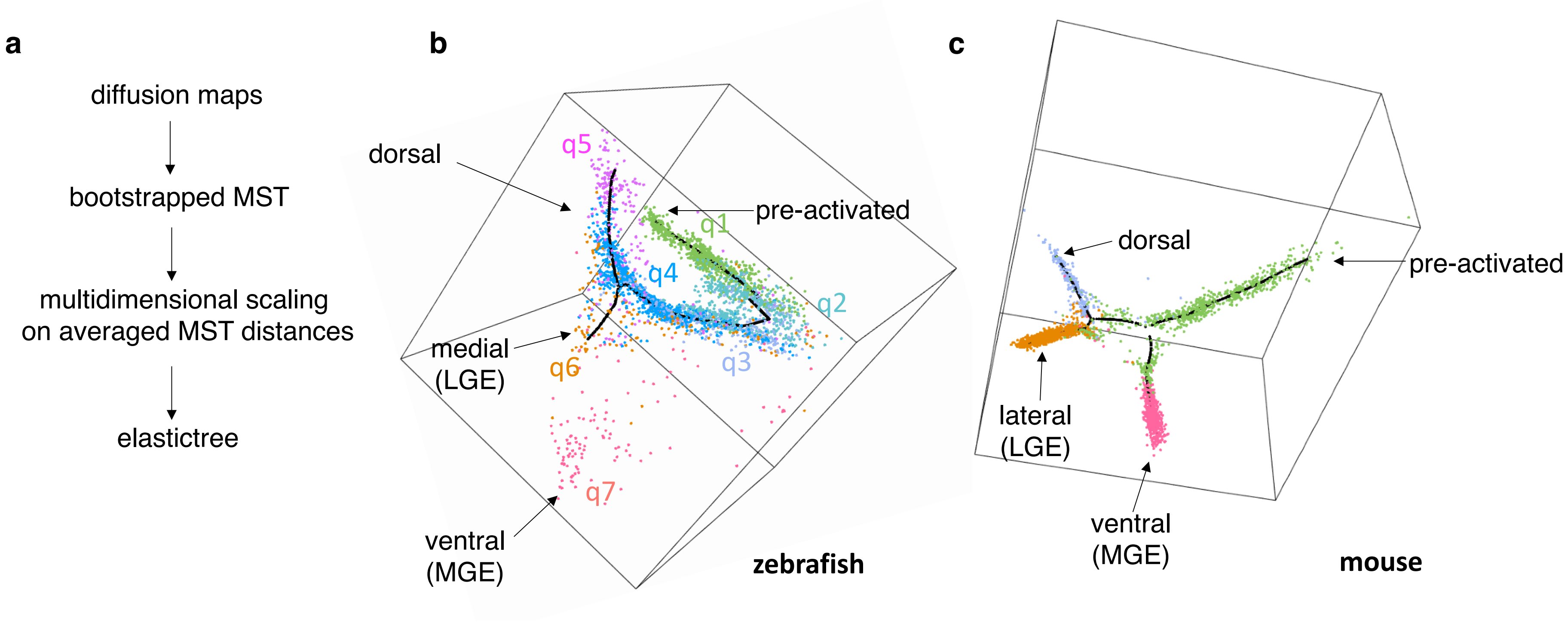

Supplementary data Fig.8. Pseudo-ordering reconstruction in zebrafish and mouse. a, Pipeline developed to determine pseudo-ordering, inspired by [97] and [98]. **b,** Annotated pseudo-ordering of qRG in the zebrafish adult telencephalon, color-coded as in Fig.2a. **c,** Annotated pseudo-ordering of qRG from the mouse SEZ from [99], colors are matched to those of zebrafish for the same regions and for pre-activated cells.

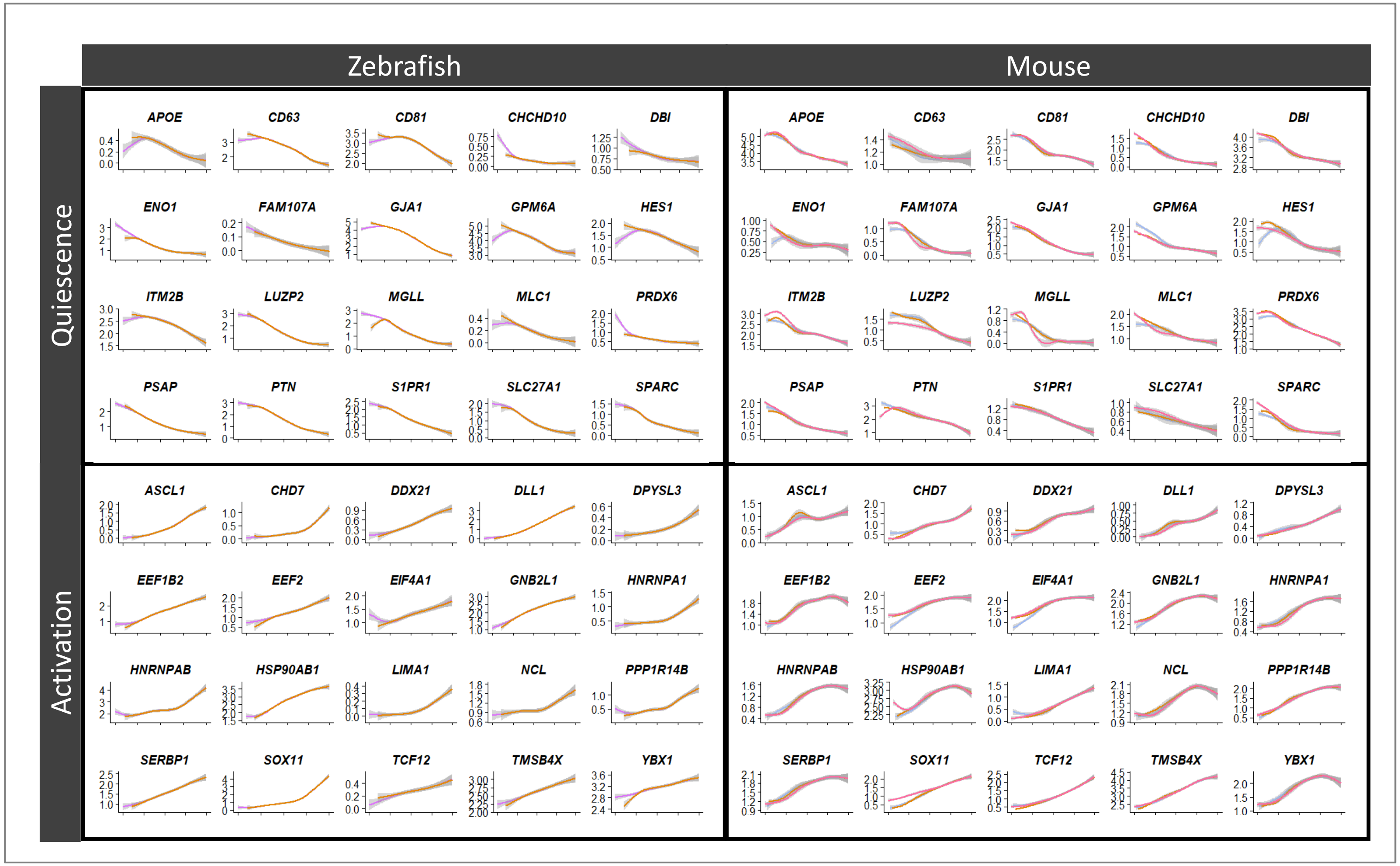

Supplementary data Fig.9. Conserved gene expression trajectories in zebrafish and mouse. Examples of genes with a conserved expression trajectory from deep quiescence to activation (from left to right on the x axis) in the adult zebrafish telencephalon and mouse SEZ. Curve colors match region of origin (see Supplementary data Fig.S8b).

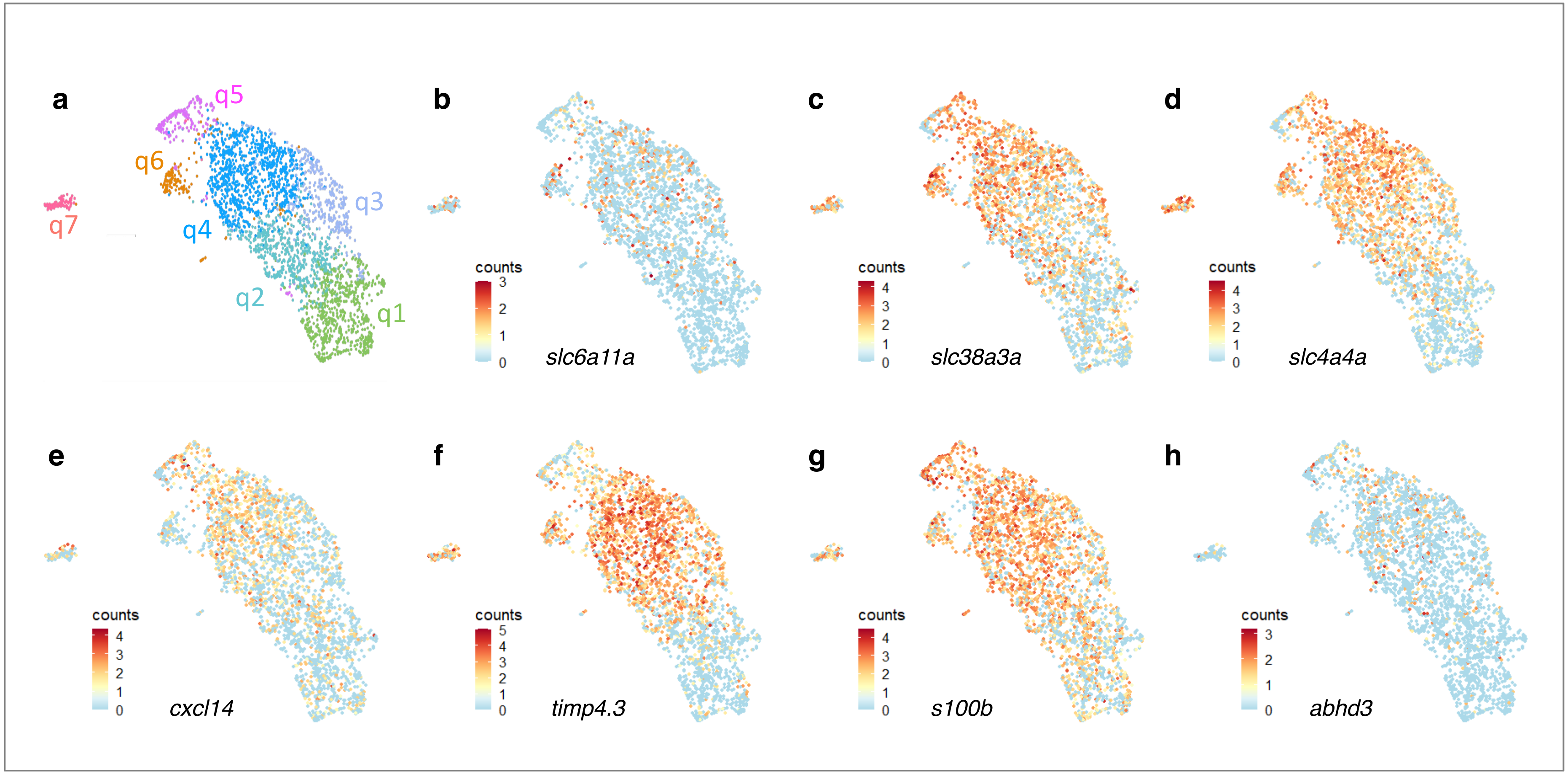

Supplementary data Fig.10. Expression of q4-enriched genes. a, Annotated UMAP of zebrafish qRG (similar to Fig.2a). **b-h,** Examples of expression of genes enriched in q4 over q2.

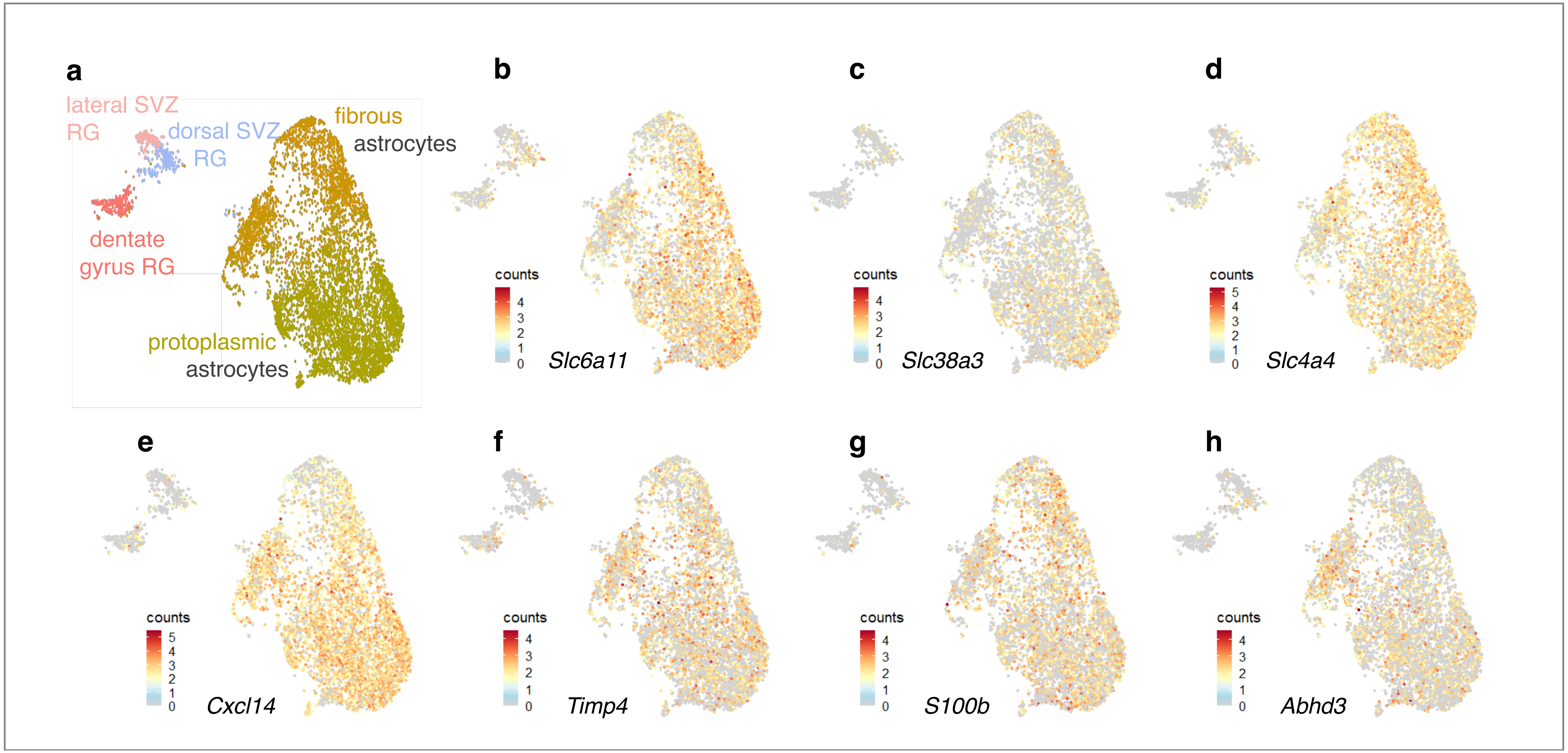

Supplementary data Fig.11. Expression of orthologs of zebrafish q4-enriched genes in the mouse telencephalon. **a,** Annotated UMAP of telencephalic astroglia from a mouse brain atlas [100]. **b-h,** Example of expression of orthologs of genes enriched in zebrafish q4 over q2 plotted in the mouse telencephalon UMAP.

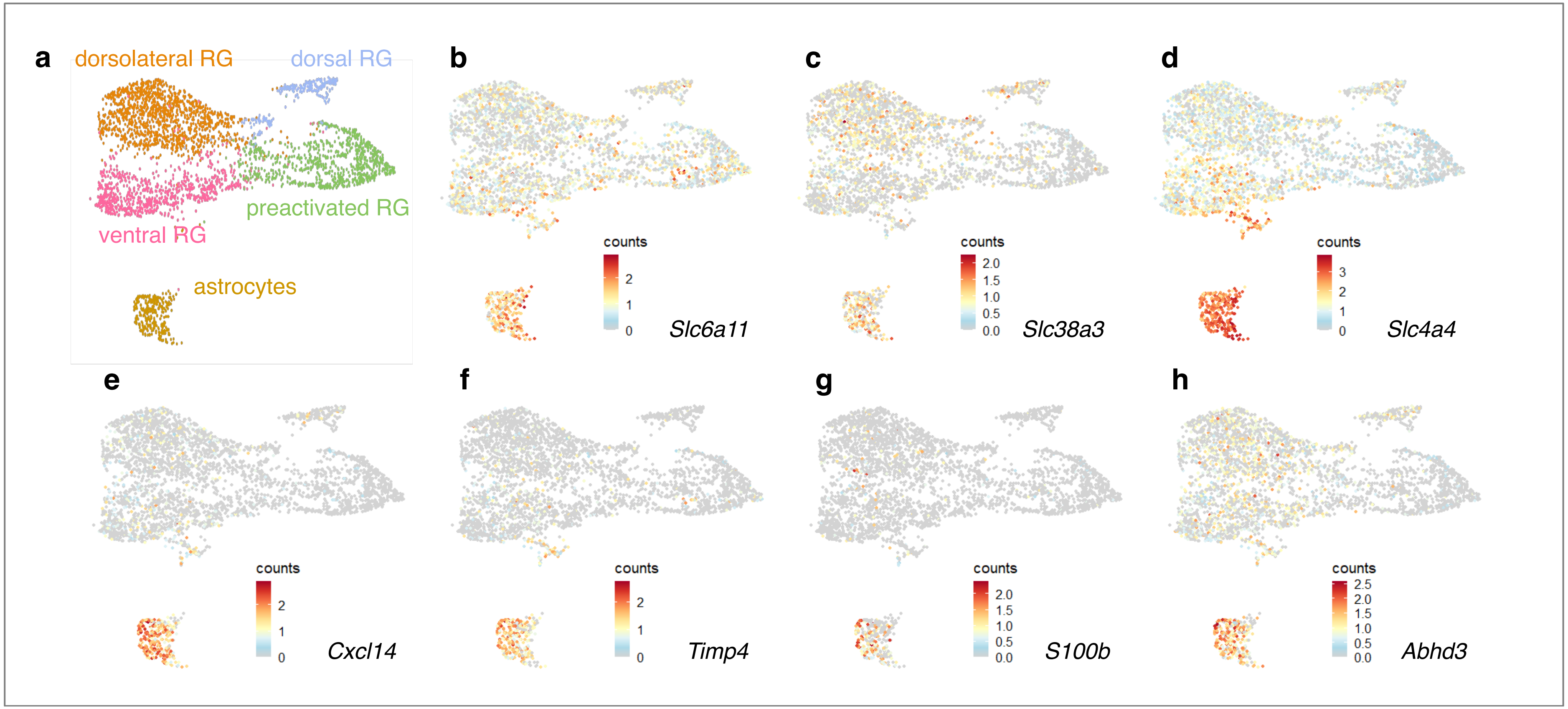

Supplementary data Fig.12. Expression of orthologs of zebrafish q4-enriched genes in the mouse SEZ. a, Annotated UMAP of telencephalic astroglia from mouse SEZ [99]. **b-h,** Examples of expression of orthologs of genes enriched in zebrafish q4 over q2 plotted in the mouse SEZ UMAP.

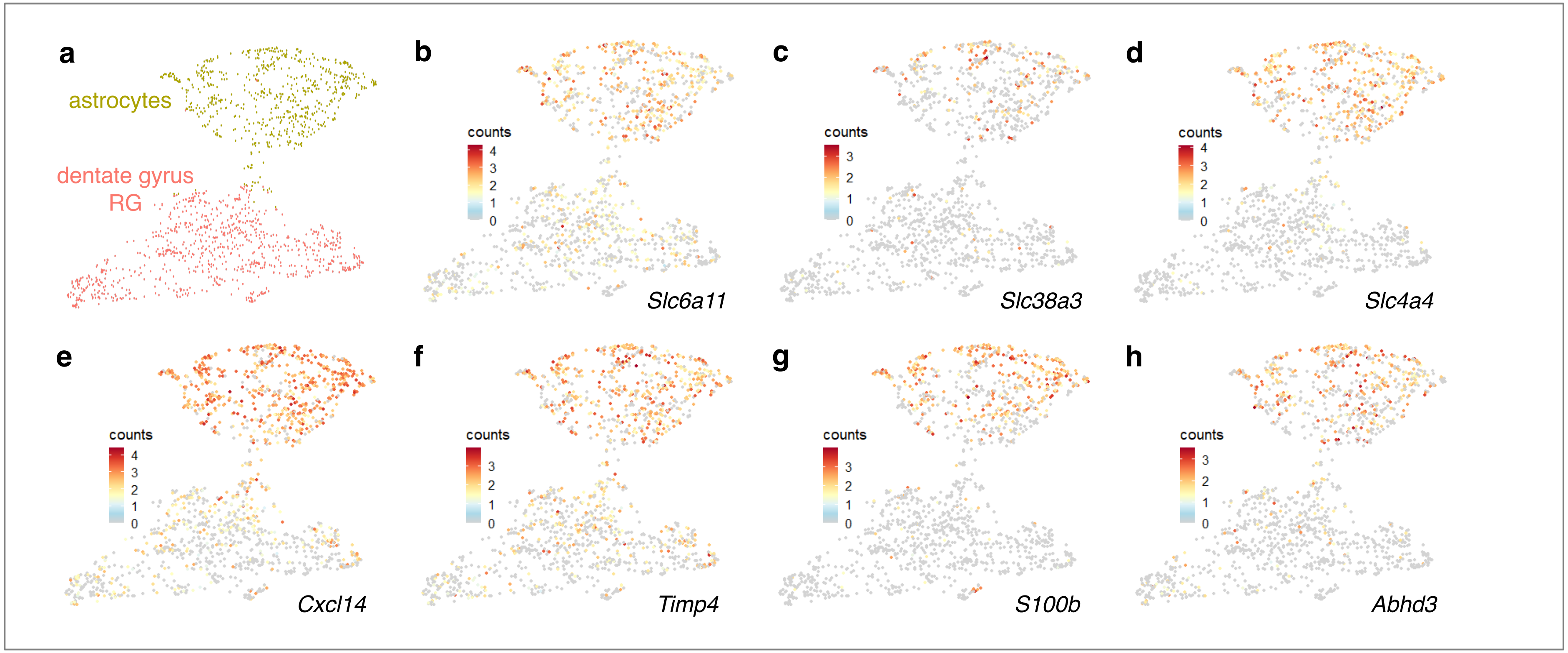

Supplementary data Fig.13. Expression of zebrafish orthologs of q4-enriched genes in the mouse hippocampus. **a,** Annotated UMAP of astroglia from mouse hippocampus [101]. **b-h,** Examples of expression of orthologs of genes enriched in zebrafish q4 over q2 plotted in the mouse hippocampus UMAP.

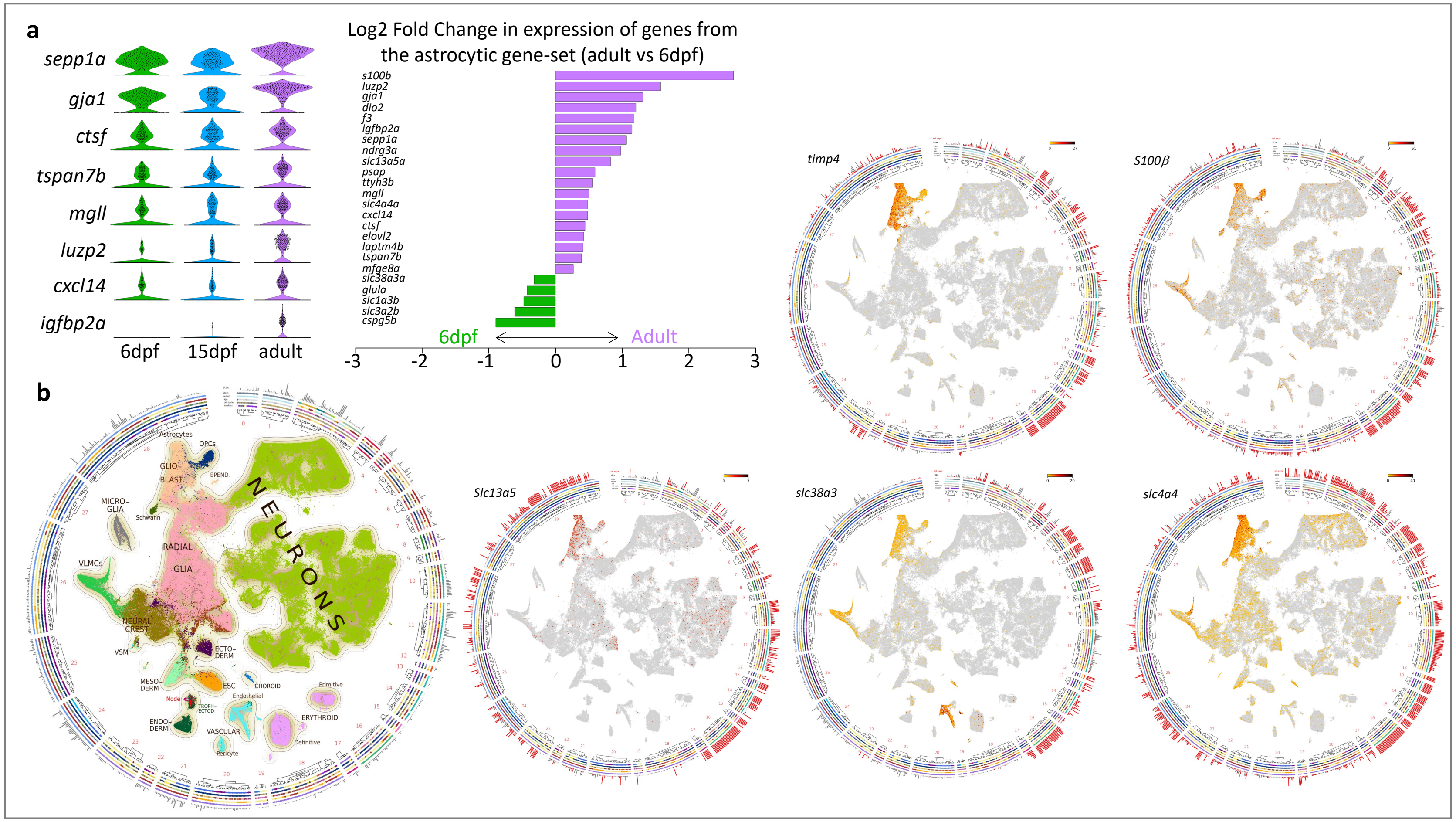

Supplementary data Fig.14. Expression of genes of the astrocytic gene set. a, Change in expression of genes from the astrocytic gene-set in zebrafish RG throughout life, based on a dataset from [102]. Left : violin plots of selected genes at 6dpf, 15dpf and in adult RG showing changes in expression as fish age; Right : barplots displaying the log2 fold change of expression of all genes from the astrocytic gene-set reliably detected at both 6dpf and in the adult. **b,** Expression of genes from the astrocytic gene-set in the developing mouse brain, showing enrichment in glioblasts which will generate parenchymal astrocytes over embryonic RGs. Plots generated from http://mousebrain.org/wheel/ using the dataset of [103].

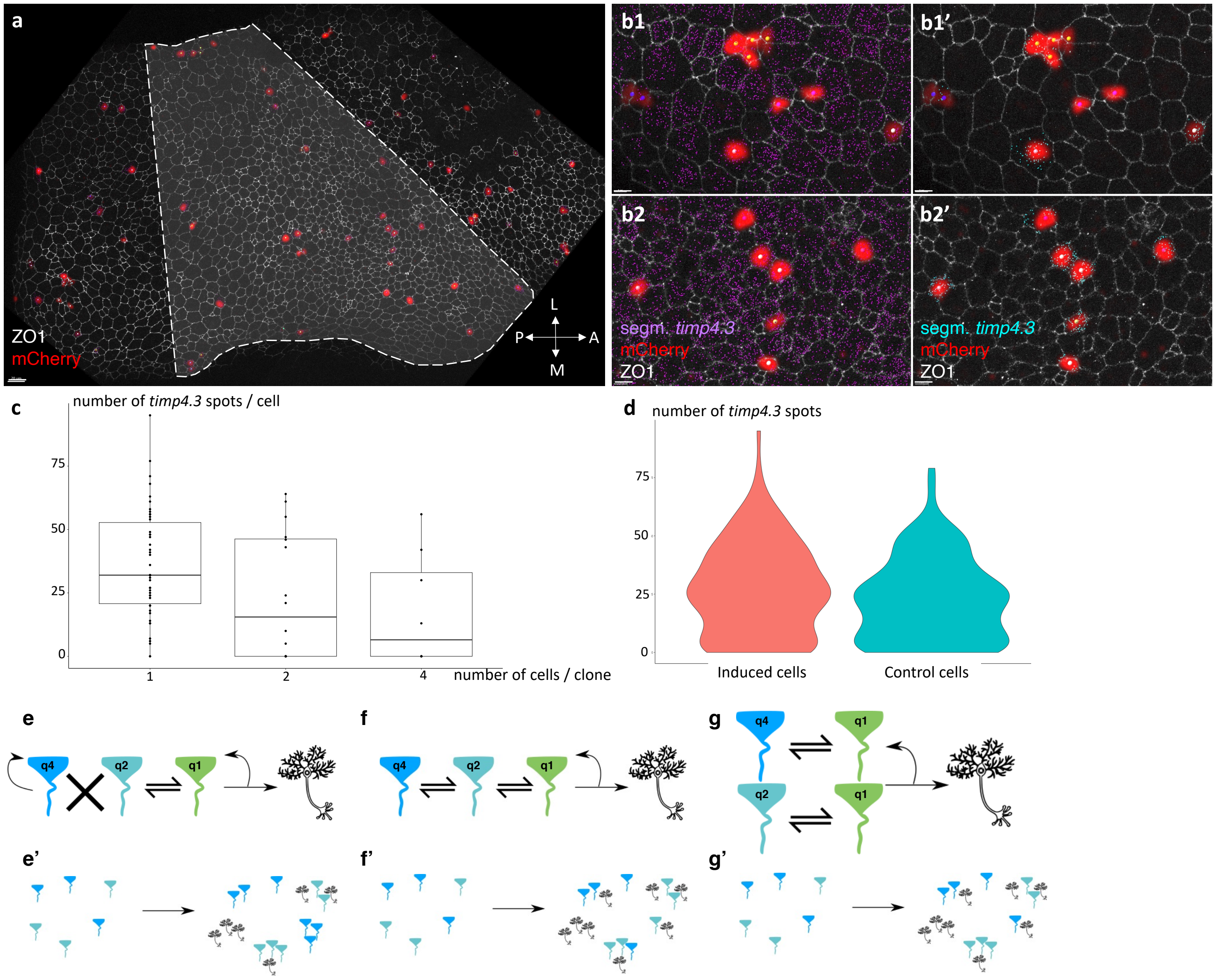
a
ZO1
mCherry
L
P
A
M
b1
b1'
b2
b2'
segm. timp4.3
mCherry
ZO1
segm. timp4.3
mCherry
ZO1
c
number of timp4.3 spots / cell
75
50
25
0
1
2
4
number of cells / clone
d
number of timp4.3 spots
75
50
25
0
Induced cells
Control cells
e
q4
q2
q1
f
q4
q2
q1
g
q4
q1
q2
q1
e'
f'
g'

Supplementary data Fig.15. Controls and rationale of the clonal analysis. a, Whole-mount view of a pallial hemisphere at 6 days-post-induction (dpi) illustrating the analyzed area (surrounded with a dotted line, same as in [104], anteroposterior and mediolateral orientations are indicated), and highlighting recombined clones (mCherry, red) and apical cell contours (ZO1, white) revealed with IHC. **b,** Higher magnifications of two example areas (1,2) showing clones (red) (central dot in cells coding the number of cells per clone at 6dpi: white, 1 cell; blue: 2 cells; yellow: 4 cells), apical cell contours, and *timp4.3* transcripts revealed by RNAscope. Left panels show all segmented *timp4.3* dots (magenta), right panels only show segmented *timp4.3* dots assigned to mCherry-positive cells (cyan). **c,** Average number of *timp4.3* dots per clone as a function of the number of cells in clones at 6dpi showing that, as expected, *timp4.3* expression decreases as cells progress along the lineage through divisions. n = 46 cells for clones containing a single cell, n = 16 cells for clones containing 2 cells, n = 8 cells for clones containing 4 cells. **d,** Bootstrapped violin plot of *timp4.3* expression levels (y axis, number of *timp4.3* dots per cell) in induced versus non-induced (control) cells, showing similar distributions. **e-g',** Hypotheses on the behavior of q4 RG (**e,f,g**) and outcome after chase (**e',f',g'**). **e,e'**: in the first scenario, q4 RG are not neurogenic but are possibly self-replicating like astrocytes. In a clonal analysis labeling both q4 and q2 RG upon induction, the number of clones that have not produced any neurons after chase should be at least equal to the number of q4 RG labeled initially. **f,f'**: in the second scenario, q4 RG represent a substate in the quiescence phase of qRG, cells can transit between q4 and q2 and participate in neurogenesis. In a clonal analysis labeling both q4 and q2 cells upon induction, the number of clones that have not given rise to neurons after chase is inferior to the number of q4 cells labeled initially. **G,G'**: in the third scenario, q4 and q2 cells are distinct subpopulations but both can activate and participate in neurogenesis. In a clonal analysis labeling both q4 and q2 cells upon induction, the number of clones that have not given rise to neurons after chase is inferior to the number of q4 cells labeled initially. Additionally, clones should be made up of only one type of qRG. Scale bars: A: 20µm; B-B2': 7µm.

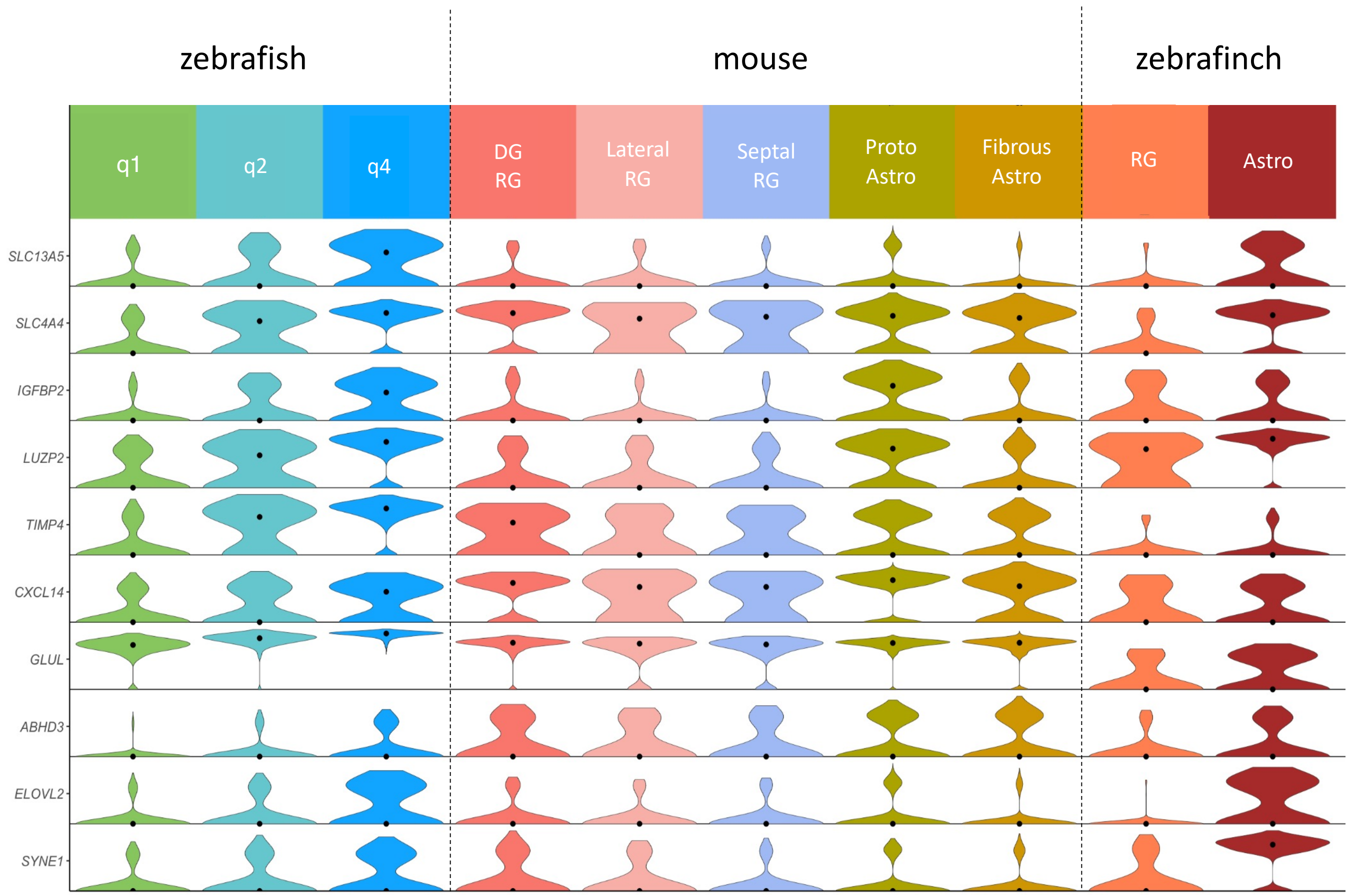

Supplementary data Fig.16. Conservation of astrocyte-enriched genes across species. Violin subset of genes detected as being enriched in q4 over q2 in zebrafish and in astrocytes over RG in mouse, plotted in zebrafish, mouse [100] and zebra finch [105]. Astrocytes from the dentate gyrus were likely not properly separated from RG of the same region, leading to an overestimation of the expression of some transcripts in DG RG here (see Supplementary data Fig.S11).

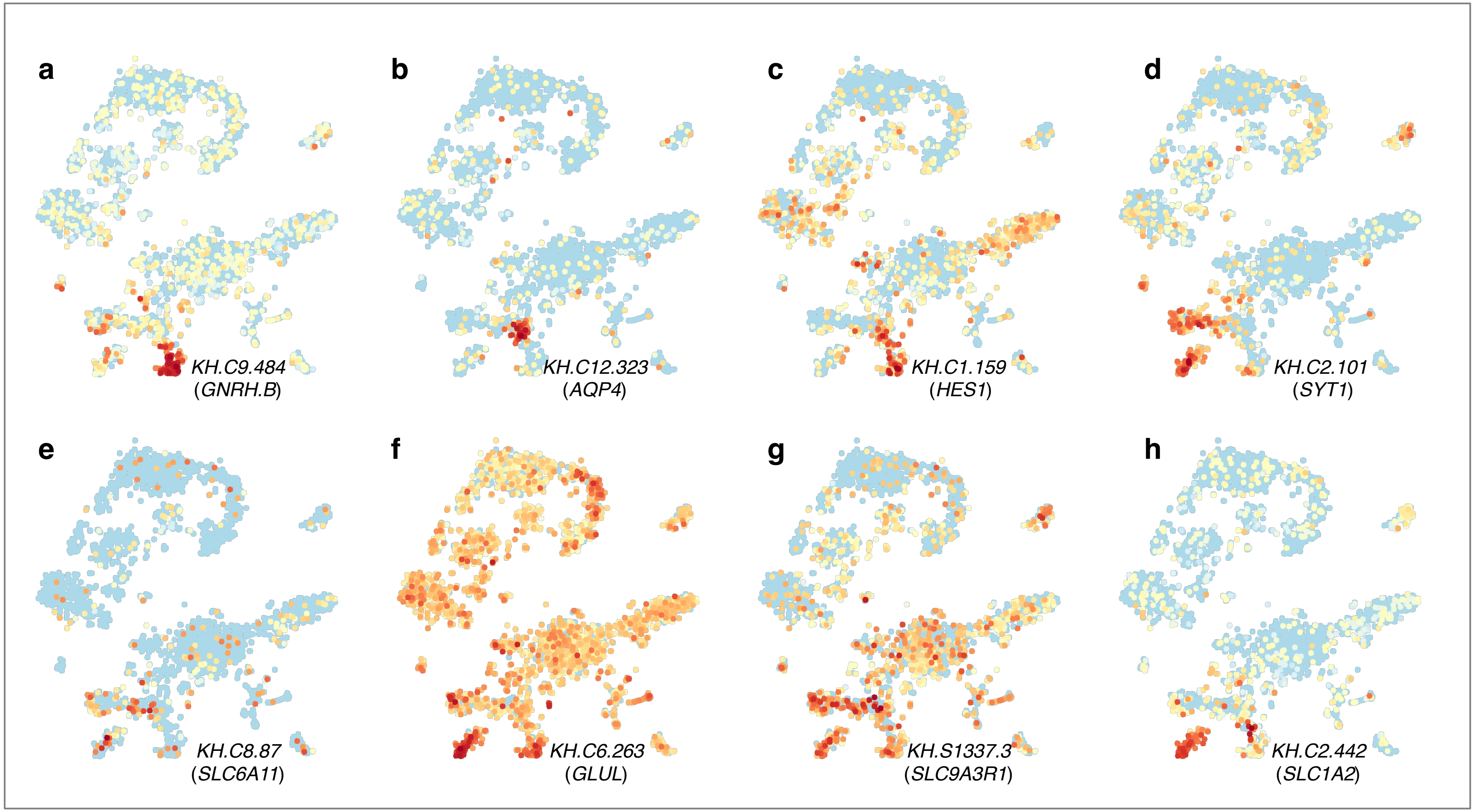

Supplementary data Fig.17. Expression of astrocytic genes in Ciona intestinalis swimming larva [106]**, plotted on a scRNAseq UMAP of ciona larval brain cells**. **a-c,** Expression of genes enriched in ciona ependymoglia (*AQP4* and *HES1* are also markers of mammalian astroglia). **d,** Expression of a neuronal marker. The cell populations identified with these markers match clusters 5 (ependymoglia) and 3 (differentiated dorsal brain) from the original publication respectively. **e-h,** Expression of genes belonging to the astrocytic synapomere. Although these genes are enriched in astroglia in vertebrates, they are expressed at higher levels in neurons than in radial ependymoglia in Ciona.

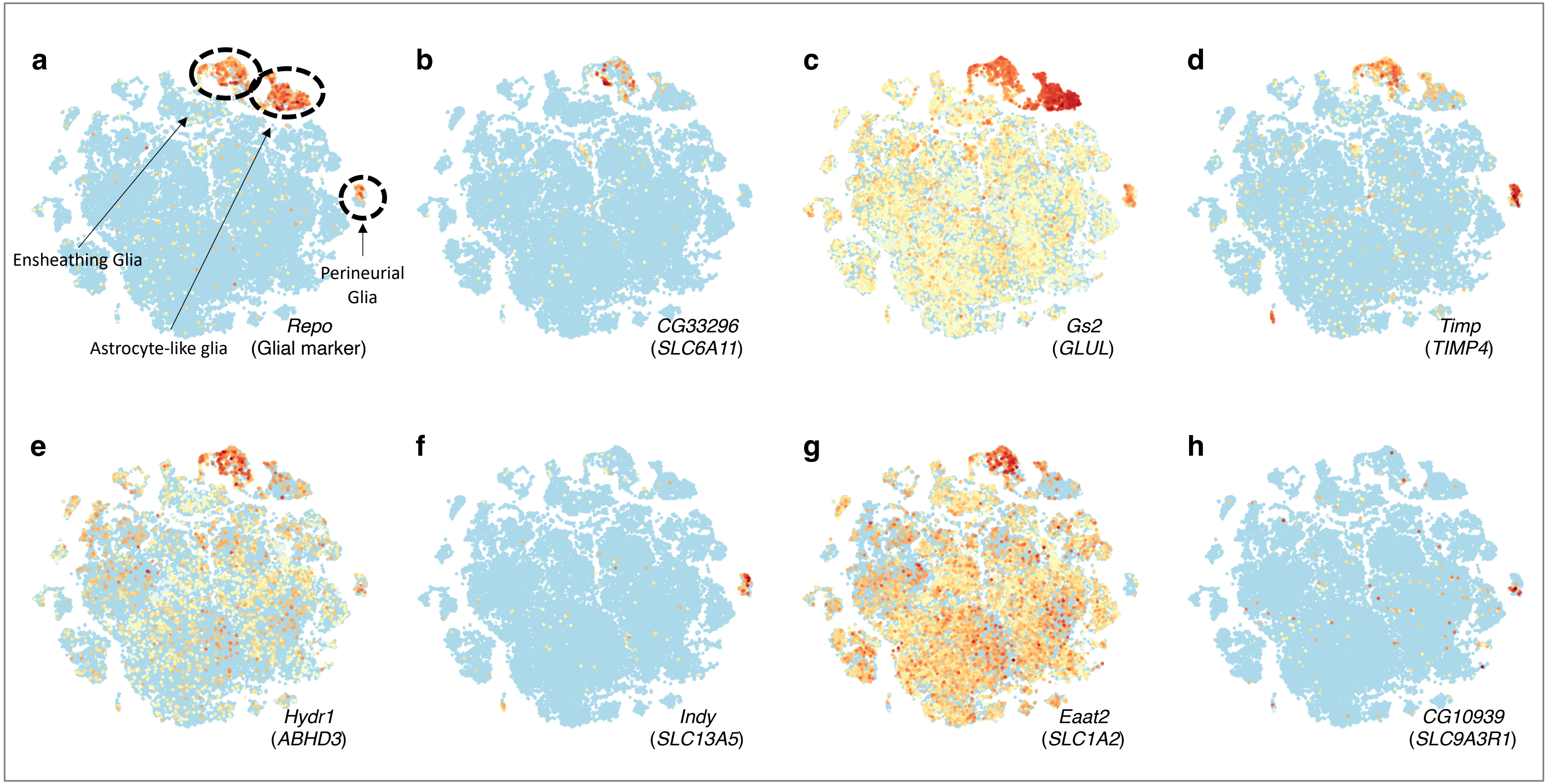

Supplementary data Fig.18. Expression of astrocytic genes in Drosophila melanogaster [107]**, plotted on a scRNAseq UMAP of cells isolated from adult Drosophila brains**. **a,** *Repo* is used as a general marker of glia, with three major subpopulations highlighted. **b-h,** Expression of orthologs for genes enriched in zebrafish q4 cells and mammalian astrocytes is then depicted, demonstrating their enrichment in ensheathing glia and their distribution across distinct glial populations.

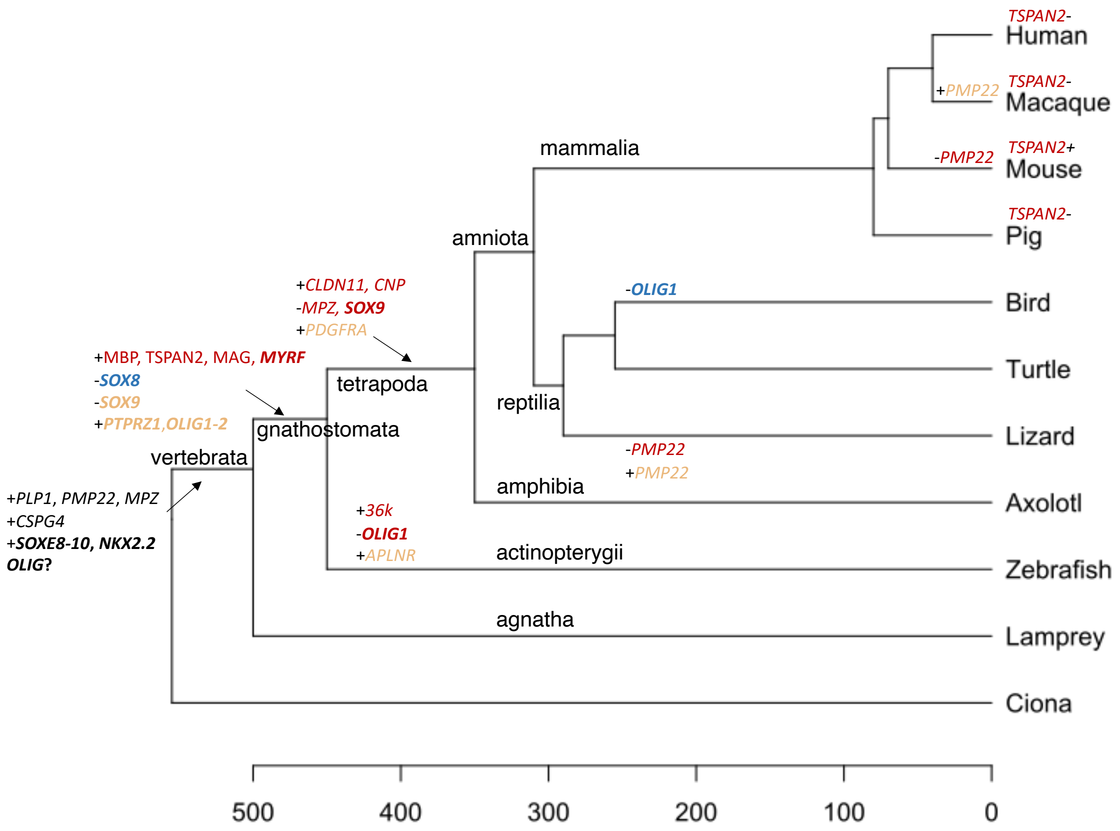

Supplementary data Fig.19. Molecular evolution of oligodendrogenesis. Phylogenetic tree annotated with changes in gene expression inferred from cross-species analysis. Light brown/beige represents changes that happened in OPCs whereas dark brown represents changes in the expression of genes in differentiating/differentiated progeny. Of note this means that some genes represented here can be expressed in daughter cells of OPCs but at different stages of maturation. Changes that happened in both OPCs and their progeny are in blue. Transcription factor-encoding genes are bolded. The expression changes that predate the split between gnathostomes and agnatha is in black without separation between OPCs and their progeny because genes are expressed in the same cell population. We could not distinguish between a loss of *TSPAN2* expression early in mammalian evolution followed by a subsequent gain in rodents or two independent losses in pig and in primates.

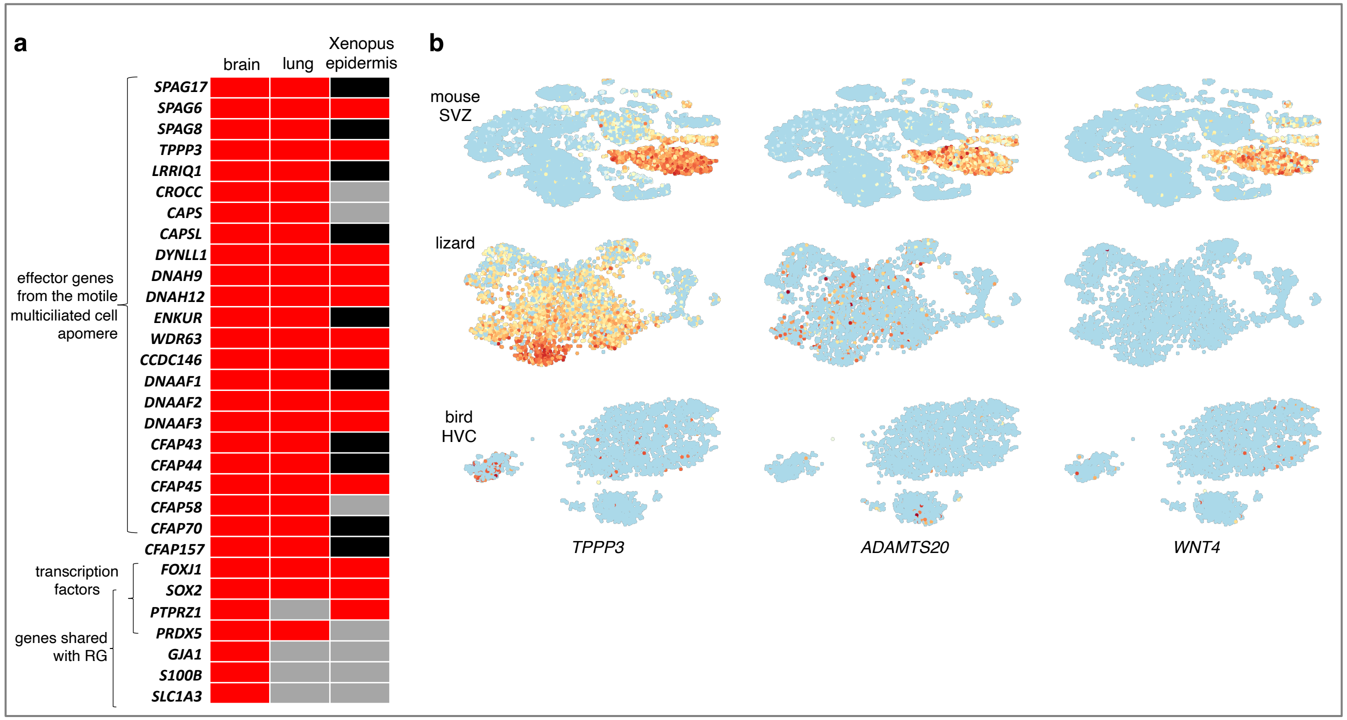

Supplementary data Fig.20. Evolution of ependymocytes. **a,** Expression of genes related to motile cilia function (top bracket) or shared with astroglia (bottom bracket) in mammalian ependymocytes, human airway ciliated cells [108] and xenopus epidermis ciliated cells [109]. Red squares indicate expression, grey squares indicate lack of expression. In xenopus, black squares indicate that the genes were not detected in any cell cluster in the dataset, which can be either true lack of expression or due to technical artifacts. The top bracket encompasses genes that form a distinctive multiciliated cell apomere that was co-opted several times to produce multiciliated cells with motile cilia in different tissues and species. Despite this convergence, ependymocytes retain exclusive shared expression of several genes with RG, and can be separated from other multiciliated cells based on a limited number of transcription factors, consistent with proposed models of cell-type evolution [110]. **b,** De novo expression of niche-modifying factors in mammalian ependymocytes. TPPP3 is used to highlight ependymocytes in three datasets: first a SEZ dataset containing all cell types in mouse, second a dataset of lizard telencephalon containing all astroglial cells and third a bird HVC dataset containing all astroglial cells (distinct astroglial cell types were too hard to distinguish on an embedding of all cells in lizard and bird because these datasets were more diverse and included a smaller proportion of astoglial cells). ADAMTS20 and WNT4 are neurogenesis regulators expressed by ependymocytes in mice but not detected in lizard nor bird ependymocytes.

SLC1A2	Glutamate clearance from the extracellular space
GLUL	Synthesis of glutamine from glutamate, participating in glutamate detoxification
CXCL14	Related to cytokines
MFGE8	Inhibition of mTOR, modulation of phagocytosis
GJA1	Gap junction
IGFBP2	Binding of insulin growth factors
CSPG5	Proteoglycan
PTN	Secreted signaling protein
SLC4A4	Bicarbonate co-transporter involved in pH regulation
S1PR1	Sphingosine receptor
S100B	Calcium binding protein
SEPP1	Extracellular antioxidant
SLC6A11	GABA uptake transporter modulating the length of Gabaergic transmission
GPM6A	
TIMP4	Metallopeptidase inhibitor controlling extracellular matrix composition
SLC1A3	Glutamate transporter involved in termination of glutamate transmission
TSPAN7	
APOE	Lipidic transporter
PTPLB	Enzyme involved in the production of very long chain fatty acids
SLC38A3	Sodium-Amino Acid co-transporter, involved in transport of glutamine and neurotransmitters
MGLL	Converts monoacylglycerides to free fatty acids and glycerol
HEPACAM	Membrane adhesion protein
ABHD3	Involved in degradation of medium chain phospholipids
LUZP2	Leucine zipper transcription factor
SLC9A3R1	Sodium/proton exchanger with a potential role in ionic and pH control
DIO2	Involved in local production of active thyroid hormone
ELOVL2	Involved in elongation of very long chain fatty acids
SYNE1	Synaptic nuclear envelope protein
SLC13A5	Sodium-citrate co transporter involved in the regulation of metabolic processes
MTSS1L	
TMEM176B	
SLC3A2	Transporter involved in calcium concentration regulation and transport of amino acids
SLC27A1	Involved in transcellular lipid transport, in particular of long chain fatty acids
TPI1	Enzyme involved in glucolysis and gluconeogenesis
ENO1	Enzyme with glycolytic activity
TTYH3	Belongs to a family of chlorine ion channels
LAPTM4B	Involved in lysosomal activity and promotion of autophagy
PHYHIPL	Predicted to be involved in fatty acid metabolism
PSAP	Involved in lysosomal activity, principally by promoting catabolism of glycosphingolipids

SPRED1	Regulator of the MAPK pathway
LRP1	Involved in lipid homeostasis
EPDR1	Predicted to be involved in calcium-mediated cell-cell interactions
ASAH1	Promotes degradation of ceramide into sphingosine and free fatty acids in lysosomes
PLXNB1	Involved in modulation of cell-cell interactions and control of cell shape
CTSF	Part of the lysosomal proteolytic system
NDRG3	
F3	

Supplementary Table 1: Genes with highest confidence of conserved enrichment in q4 over q2 and in astrocytes over RG. Gene names are in the left column and known functions pertaining to a potential role in the brain are mentioned in the right column. Genes in bold have been used to label astrocytes in the past with genes in red having been used as distinctive markers between astrocytes and RG in mammalian brains.

Supplementary references

1. Fiorelli, R., Azim, K., Fischer, B. & Raineteau, O. Adding a spatial dimension to postnatal ventricular-subventricular zone neurogenesis. *Development* **142**, 2109–2120 (2015).
2. Merkle, F. T., Mirzadeh, Z. & Alvarez-Buylla, A. Mosaic Organization of Neural Stem Cells in the Adult Brain. *Science* **317**, 381–384 (2007).
3. Young, K. M., Fogarty, M., Kessaris, N. & Richardson, W. D. Subventricular Zone Stem Cells Are Heterogeneous with Respect to Their Embryonic Origins and Neurogenic Fates in the Adult Olfactory Bulb. *J. Neurosci.* **27**, 8286–8296 (2007).
4. Alvarez-Buylla, A., Kohwi, M., Nguyen, T. M. & Merkle, F. T. The Heterogeneity of Adult Neural Stem Cells and the Emerging Complexity of Their Niche. *Cold Spring Harb Symp Quant Biol* **73**, 357–365 (2008).
5. Ihrie, R. A. *et al.* Persistent Sonic Hedgehog Signaling in Adult Brain Determines Neural Stem Cell Positional Identity. *Neuron* **71**, 250–262 (2011).
6. Giachino, C. *et al.* Molecular Diversity Subdivides the Adult Forebrain Neural Stem Cell Population. *Stem Cells* **32**, 70–84 (2014).
7. Fuentealba, L. C. *et al.* Embryonic Origin of Postnatal Neural Stem Cells. *Cell* **161**, 1644–1655 (2015).
8. Zweifel, S. *et al.* HOPX Defines Heterogeneity of Postnatal Subventricular Zone Neural Stem Cells. *Stem Cell Reports* **11**, 770–783 (2018).
9. Delgado, R. N. *et al.* Maintenance of neural stem cell positional identity by mixed-lineage leukemia 1. *Science* **368**, 48–53 (2020).
10. Marcy, G. *et al.* Single cell analysis of the dorsal V-SVZ reveals differential quiescence of postnatal pallial and subpallial neural stem cells driven by TGFbeta/BMP-signalling. 2022.05.20.492790 Preprint at https://doi.org/10.1101/2022.05.20.492790 (2022).
11. Wullimann, M. F., Rupp, B. & Reichert, H. *Neuroanatomy of the Zebrafish Brain*. (Birkhäuser, Basel, 1996). doi:10.1007/978-3-0348-8979-7.

12. Cosacak, M. I. *et al.* Single-Cell Transcriptomics Analyses of Neural Stem Cell Heterogeneity and Contextual Plasticity in a Zebrafish Brain Model of Amyloid Toxicity. *Cell Reports* **27**, 1307-1318.e3 (2019).
13. März, M. *et al.* Heterogeneity in progenitor cell subtypes in the ventricular zone of the zebrafish adult telencephalon. *Glia* **58**, 870–888 (2010).
14. Grandel, H., Kaslin, J., Ganz, J., Wenzel, I. & Brand, M. Neural stem cells and neurogenesis in the adult zebrafish brain: Origin, proliferation dynamics, migration and cell fate. *Developmental Biology* **295**, 263–277 (2006).
15. Rupprecht, P. & Friedrich, R. W. Precise Synaptic Balance in the Zebrafish Homolog of Olfactory Cortex. *Neuron* **100**, 669-683.e5 (2018).
16. Tosches, M. A. *et al.* Evolution of pallium, hippocampus, and cortical cell types revealed by single-cell transcriptomics in reptiles. *Science* **360**, 881–888 (2018).
17. Lust, K. *et al.* Single-cell analyses of axolotl forebrain organization, neurogenesis, and regeneration. 2022.03.21.485045 Preprint at https://doi.org/10.1101/2022.03.21.485045 (2022).
18. Woych, J. *et al.* Cell type profiling in salamanders identifies innovations in vertebrate forebrain evolution. 2022.03.28.485354 Preprint at https://doi.org/10.1101/2022.03.28.485354 (2022).
19. Dirian, L. *et al.* Spatial Regionalization and Heterochrony in the Formation of Adult Pallial Neural Stem Cells. *Developmental Cell* **30**, 123–136 (2014).
20. Rodríguez, F. *et al.* Spatial Cognition in Teleost Fish: Strategies and Mechanisms. *Animals* **11**, 2271 (2021).
21. Gómez, A., Rodríguez-Expósito, B., Ocaña, F. M., Salas, C. & Rodríguez, F. Trace classical conditioning impairment after lesion of the lateral part of the goldfish telencephalic pallium suggests a long ancestry of the episodic memory function of the vertebrate hippocampus. *Brain Struct Funct* **227**, 2879–2890 (2022).
22. Norimoto, H. *et al.* A claustrum in reptiles and its role in slow-wave sleep. *Nature* **578**, 413–418 (2020).
23. Schede, H. H. *et al.* Spatial tissue profiling by imaging-free molecular tomography. *Nat Biotechnol* **39**, 968–977 (2021).
24. Kempermann, G. Adult Neurogenesis: An Evolutionary Perspective. *Cold Spring Harb Perspect Biol* **8**, a018986 (2016).
25. Powers, A. S. Adult Neurogenesis in Mammals and Nonmammals: Commentary on Kempermann G (2012): New neurons for 'survival of the fittest'. Nat Rev Neurosci 13:727-736. *Brain Behavior and Evolution* **81**, 206–208 (2013).
26. Sorrells, S. F. *et al.* Human hippocampal neurogenesis drops sharply in children to undetectable levels in adults. *Nature* **555**, 377–381 (2018).
27. Boldrini, M. *et al.* Human Hippocampal Neurogenesis Persists throughout Aging. *Cell Stem Cell* **22**, 589-599.e5 (2018).
28. Cipriani, S. *et al.* Hippocampal Radial Glial Subtypes and Their Neurogenic Potential in Human Fetuses and Healthy and Alzheimer's Disease Adults. *Cerebral Cortex* **28**, 2458–2478 (2018).
29. Moreno-Jiménez, E. P. *et al.* Adult hippocampal neurogenesis is abundant in neurologically healthy subjects and drops sharply in patients with Alzheimer's disease. *Nat Med* **25**, 554–560 (2019).
30. Lucassen, P. J., Fitzsimons, C. P., Salta, E. & Maletic-Savatic, M. Adult neurogenesis, human after all (again): Classic, optimized, and future approaches. *Behavioural Brain Research* **381**, 112458 (2020).
31. Sorrells, S. F. *et al.* Positive Controls in Adults and Children Support That Very Few, If Any, New Neurons Are Born in the Adult Human Hippocampus. *J. Neurosci.* **41**, 2554–2565 (2021).
32. Terreros-Roncal, J. *et al.* Impact of neurodegenerative diseases on human adult hippocampal neurogenesis. *Science* **374**, 1106–1113 (2021).
33. Zhou, Y. *et al.* Molecular landscapes of human hippocampal immature neurons across lifespan. *Nature* **607**, 527–533 (2022).
34. Ayhan, F. *et al.* Resolving cellular and molecular diversity along the hippocampal anterior-to-posterior axis in humans. *Neuron* **109**, 2091-2105.e6

(2021).
35. Franjic, D. *et al.* Transcriptomic taxonomy and neurogenic trajectories of adult human, macaque, and pig hippocampal and entorhinal cells. *Neuron* **110**, 452-469.e14 (2022).
36. Hao, Z.-Z. *et al.* Single-cell transcriptomics of adult macaque hippocampus reveals neural precursor cell populations. *Nat Neurosci* **25**, 805–817 (2022).
37. Habib, N. *et al.* Massively parallel single-nucleus RNA-seq with DroNc-seq. *Nat Methods* **14**, 955–958 (2017).
38. Zhang, H. *et al.* Single-nucleus transcriptomic landscape of primate hippocampal aging. *Protein Cell* **12**, 695–716 (2021).
39. Rotheneichner, P. *et al.* Cellular Plasticity in the Adult Murine Piriform Cortex: Continuous Maturation of Dormant Precursors Into Excitatory Neurons. *Cerebral Cortex* **28**, 2610–2621 (2018).
40. Sorrells, S. F. *et al.* Immature excitatory neurons develop during adolescence in the human amygdala. *Nat Commun* **10**, 2748 (2019).
41. La Rosa, C. *et al.* Phylogenetic variation in cortical layer II immature neuron reservoir of mammals. *eLife* **9**, e55456 (2020).
42. Bond, A. M., Ming, G. & Song, H. What Is the Relationship Between Hippocampal Neurogenesis Across Different Stages of the Lifespan? *Frontiers in Neuroscience* **16**, (2022).
43. Tarashansky, A. J. *et al.* Mapping single-cell atlases throughout Metazoa unravels cell type evolution. *eLife* **10**, e66747 (2021).
44. Fiddes, I. T. *et al.* Human-Specific NOTCH2NL Genes Affect Notch Signaling and Cortical Neurogenesis. *Cell* **173**, 1356-1369.e22 (2018).
45. Suzuki, I. K. *et al.* Human-Specific NOTCH2NL Genes Expand Cortical Neurogenesis through Delta/Notch Regulation. *Cell* **173**, 1370-1384.e16 (2018).
46. Dang, L., Yoon, K., Wang, M. & Gaiano, N. Notch3 Signaling Promotes Radial Glial/Progenitor Character in the Mammalian Telencephalon. *DNE* **28**, 58–69 (2006).
47. Fiddes, I. T., Pollen, A. A., Davis, J. M. & Sikela, J. M. Paired involvement of human-specific Olduvai domains and NOTCH2NL genes in human brain evolution. *Hum Genet* **138**, 715–721 (2019).
48. Gould, E. *et al.* Hippocampal neurogenesis in adult Old World primates. *Proceedings of the National Academy of Sciences* **96**, 5263–5267 (1999).
49. Kornack, D. R. & Rakic, P. Continuation of neurogenesis in the hippocampus of the adult macaque monkey. *Proceedings of the National Academy of Sciences* **96**, 5768–5773 (1999).
50. Pencea, V., Bingaman, K. D., Freedman, L. J. & Luskin, M. B. Neurogenesis in the Subventricular Zone and Rostral Migratory Stream of the Neonatal and Adult Primate Forebrain. *Experimental Neurology* **172**, 1–16 (2001).
51. Duque, A., Arellano, J. I. & Rakic, P. An assessment of the existence of adult neurogenesis in humans and value of its rodent models for neuropsychiatric diseases. *Mol Psychiatry* **27**, 377–382 (2022).
52. Niimura, Y., Matsui, A. & Touhara, K. Acceleration of Olfactory Receptor Gene Loss in Primate Evolution: Possible Link to Anatomical Change in Sensory Systems and Dietary Transition. *Molecular Biology and Evolution* **35**, 1437–1450 (2018).
53. Reep, R. L., Finlay, B. L. & Darlington, R. B. The Limbic System in Mammalian Brain Evolution. *Brain Behavior and Evolution* **70**, 57–70 (2007).
54. Aboitiz, F. & Montiel, J. F. Olfaction, navigation, and the origin of isocortex. *Frontiers in Neuroscience* **9**, (2015).
55. Zhang, R. *et al.* Id4 Downstream of Notch2 Maintains Neural Stem Cell Quiescence in the Adult Hippocampus. *Cell Reports* **28**, 1485-1498.e6 (2019).
56. Ge, W. *et al.* Notch signaling promotes astrogliogenesis via direct CSL-mediated glial gene activation. *Journal of Neuroscience Research* **69**, 848–860 (2002).
57. Hu, X., He, W., Luo, X., Tsubota, K. E. & Yan, R. BACE1 Regulates Hippocampal Astrogenesis via the Jagged1-Notch Pathway. *Cell Reports* **4**, 40–

49 (2013).
58. Chambers, C. B. *et al.* Spatiotemporal selectivity of response to Notch1 signals in mammalian forebrain precursors. *Development* **128**, 689–702 (2001).
59. Grandbarbe, L. *et al.* Delta-Notch signaling controls the generation of neurons/glia from neural stem cells in a stepwise process. *Development* **130**, 1391–1402 (2003).
60. Namihira, M. *et al.* Committed Neuronal Precursors Confer Astrocytic Potential on Residual Neural Precursor Cells. *Developmental Cell* **16**, 245–255 (2009).
61. Manganas, L. N. *et al.* Magnetic Resonance Spectroscopy Identifies Neural Progenitor Cells in the Live Human Brain. *Science* **318**, 980–985 (2007).
62. Hartline, D. K. The evolutionary origins of glia. *Glia* **59**, 1215–1236 (2011).
63. Rey, S., Zalc, B. & Klämbt, C. Evolution of glial wrapping: A new hypothesis. *Developmental Neurobiology* **81**, 453–463 (2021).
64. Yoshimura, S., Murray, J. I., Lu, Y., Waterston, R. H. & Shaham, S. mls-2 and vab-3 control glia development, hlh-17/Olig expression and glia-dependent neurite extension in C. elegans. *Development* **135**, 2263–2275 (2008).
65. Bacaj, T., Tevlin, M., Lu, Y. & Shaham, S. Glia Are Essential for Sensory Organ Function in C. elegans. *Science* **322**, 744–747 (2008).
66. Wilson, C. H. & Hartline, D. K. Novel organization and development of copepod myelin. i. ontogeny. *Journal of Comparative Neurology* **519**, 3259–3280 (2011).
67. Kálmán, M., Matuz, V., Sebők, O. M. & Lőrincz, D. Evolutionary Modifications Are Moderate in the Astroglial System of Actinopterygii as Revealed by GFAP Immunohistochemistry. *Frontiers in Neuroanatomy* **15**, (2021).
68. Jurisch-Yaksi, N., Yaksi, E. & Kizil, C. Radial glia in the zebrafish brain: Functional, structural, and physiological comparison with the mammalian glia. *Glia* **68**, 2451–2470 (2020).
69. Chen, J., Poskanzer, K. E., Freeman, M. R. & Monk, K. R. Live-imaging of astrocyte morphogenesis and function in zebrafish neural circuits. *Nat Neurosci* **23**, 1297–1306 (2020).
70. Mu, Y. *et al.* Glia Accumulate Evidence that Actions Are Futile and Suppress Unsuccessful Behavior. *Cell* **178**, 27-43.e19 (2019).
71. Gebara, E. *et al.* Heterogeneity of Radial Glia-Like Cells in the Adult Hippocampus. *Stem Cells* **34**, 997–1010 (2016).
72. Asrican, B. *et al.* Neuropeptides Modulate Local Astrocytes to Regulate Adult Hippocampal Neural Stem Cells. *Neuron* **108**, 349-366.e6 (2020).
73. Bao, H. *et al.* Long-Range GABAergic Inputs Regulate Neural Stem Cell Quiescence and Control Adult Hippocampal Neurogenesis. *Cell Stem Cell* **21**, 604-617.e5 (2017).
74. Song, J. *et al.* Neuronal circuitry mechanism regulating adult quiescent neural stem-cell fate decision. *Nature* **489**, 150–154 (2012).
75. Yeh, C.-Y. *et al.* Mossy Cells Control Adult Neural Stem Cell Quiescence and Maintenance through a Dynamic Balance between Direct and Indirect Pathways. *Neuron* **99**, 493-510.e4 (2018).
76. Song, J. *et al.* Parvalbumin interneurons mediate neuronal circuitry–neurogenesis coupling in the adult hippocampus. *Nat Neurosci* **16**, 1728–1730 (2013).
77. Li, Y.-D. *et al.* Hypothalamic modulation of adult hippocampal neurogenesis in mice confers activity-dependent regulation of memory and anxiety-like behavior. *Nat Neurosci* **25**, 630–645 (2022).
78. Paul, A., Chaker, Z. & Doetsch, F. Hypothalamic regulation of regionally distinct adult neural stem cells and neurogenesis. *Science* **356**, 1383–1386 (2017).
79. Mikhailov, K. V. *et al.* The origin of Metazoa: a transition from temporal to spatial cell differentiation. *BioEssays* **31**, 758–768 (2009).

80. Brunet, T. *et al.* A flagellate-to-amoeboid switch in the closest living relatives of animals. *eLife* **10**, e61037 (2021).
81. Reichenbach, A., Neumann, M. & Brückner, G. Cell length to diameter relation of rat fetal radial glia — Does impaired K+ transport capacity of long thin cells cause their perinatal transformation into multipolar astrocytes? *Neuroscience Letters* **73**, 95–100 (1987).
82. Kálmán, M. & Pritz, M. B. Glial fibrillary acidic protein-immunopositive structures in the brain of a Crocodilian, Caiman crocodilus, and its bearing on the evolution of astroglia. *Journal of Comparative Neurology* **431**, 460–480 (2001).
83. Mugnaini, E. Cell junctions of astrocytes, ependyma, and related cells in the mammalian central nervous system, with emphasis on the hypothesis of a generalized functional syncytium of supporting cells. in *Astrocytes* (eds. Fedoroff, S. & Vernadakis, A.) 329–371 (Academic Press, 1986). doi:10.1016/B978-0-12-250451-8.50018-7.
84. Kálmán, M. & Gould, R. M. GFAP-immunopositive structures in spiny dogfish, Squalus acanthias, and little skate, Raiaerinacea, brains: differences have evolutionary implications. *Anat Embryol* **204**, 59–80 (2001).
85. Wicht, H., Derouiche, A. & Korf, H.-W. An immunocytochemical investigation of glial morphology in the Pacific hagfish: radial and astrocyte-like glia have the same phylogenetic age. *J Neurocytol* **23**, 565–576 (1994).
86. Goldman, S. A. & Nottebohm, F. Neuronal production, migration, and differentiation in a vocal control nucleus of the adult female canary brain. *Proceedings of the National Academy of Sciences* **80**, 2390–2394 (1983).
87. Alvarez-Buylla, A., Theelen, M. & Nottebohm, F. Birth of projection neurons in the higher vocal center of the canary forebrain before, during, and after song learning. *Proceedings of the National Academy of Sciences* **85**, 8722–8726 (1988).
88. Lamanna, F. *et al.* Reconstructing the ancestral vertebrate brain using a lamprey neural cell type atlas. 2022.02.28.482278 Preprint at https://doi.org/10.1101/2022.02.28.482278 (2022).
89. Weil, M.-T. *et al.* Axonal Ensheathment in the Nervous System of Lamprey: Implications for the Evolution of Myelinating Glia. *J. Neurosci.* **38**, 6586–6596 (2018).
90. Lambeth, L. & Blunt, M. J. Electron-microscopic study of monotreme neuroglia. *Acta Anat (Basel)* **93**, 155–125 (1975).
91. Kuratani, S. & Ota, K. G. Hagfish (cyclostomata, vertebrata): Searching for the ancestral developmental plan of vertebrates. *BioEssays* **30**, 167–172 (2008).
92. Sugahara, F., Murakami, Y., Pascual-Anaya, J. & Kuratani, S. Reconstructing the ancestral vertebrate brain. *Development, Growth & Differentiation* **59**, 163–174 (2017).
93. Murat, F. *et al.* The molecular evolution of spermatogenesis across mammals. 2021.11.08.467712 Preprint at https://doi.org/10.1101/2021.11.08.467712 (2021).
94. Styfhals, R. *et al.* Cell type diversity in a developing octopus brain. 2022.01.24.477459 Preprint at https://doi.org/10.1101/2022.01.24.477459 (2022).
95. Jones, B. W., Fetter, R. D., Tear, G. & Goodman, C. S. glial cells missing: a genetic switch that controls glial versus neuronal fate. *Cell* **82**, 1013–1023 (1995).
96. Belenguer, G. *et al.* Adult Neural Stem Cells Are Alerted by Systemic Inflammation through TNF-α Receptor Signaling. *Cell Stem Cell* **28**, 285-299.e9 (2021).
97. Mayer, C. *et al.* Developmental diversification of cortical inhibitory interneurons. *Nature* **555**, 457–462 (2018).
98. Shin, J. *et al.* Single-Cell RNA-Seq with Waterfall Reveals Molecular Cascades underlying Adult Neurogenesis. *Cell Stem Cell* **17**, 360–372 (2015).
99. Cebrian-Silla, A. *et al.* Single-cell analysis of the ventricular-subventricular zone reveals signatures of dorsal and ventral adult neurogenesis. *eLife* **10**,

e67436 (2021).
100. Zeisel, A. *et al.* Molecular Architecture of the Mouse Nervous System. *Cell* **174**, 999-1014.e22 (2018).
101. Harris, L. *et al.* Coordinated changes in cellular behavior ensure the lifelong maintenance of the hippocampal stem cell population. *Cell Stem Cell* **28**, 863-876.e6 (2021).
102. Pandey, S., Moyer, A. J. & Thyme, S. B. A single-cell transcriptome atlas of the maturing zebrafish telencephalon. *Genome Res* **33**, 658–671 (2023).
103. La Manno, G. *et al.* Molecular architecture of the developing mouse brain. *Nature* **596**, 92–96 (2021).
104. Than-Trong, E. *et al.* Lineage hierarchies and stochasticity ensure the long-term maintenance of adult neural stem cells. *Sci Adv* **6**, eaaz5424 (2020).
105. Colquitt, B. M., Merullo, D. P., Konopka, G., Roberts, T. F. & Brainard, M. S. Cellular transcriptomics reveals evolutionary identities of songbird vocal circuits. *Science* **371**, eabd9704 (2021).
106. Sharma, S., Wang, W. & Stolfi, A. Single-cell transcriptome profiling of the Ciona larval brain. *Developmental Biology* **448**, 226–236 (2019).
107. Davie, K. *et al.* A Single-Cell Transcriptome Atlas of the Aging Drosophila Brain. *Cell* **174**, 982-998.e20 (2018).
108. Okuda, K. *et al.* Secretory Cells Dominate Airway CFTR Expression and Function in Human Airway Superficial Epithelia. *Am J Respir Crit Care Med* **203**, 1275–1289 (2021).
109. Briggs, J. A. *et al.* The dynamics of gene expression in vertebrate embryogenesis at single-cell resolution. *Science* **360**, eaar5780 (2018).
110. Arendt, D. *et al.* The origin and evolution of cell types. *Nat Rev Genet* **17**, 744–757 (2016).
