# Supplementary material for: Reconstruction of macroglia and adult neurogenesis evolution through cross-species single-cell transcriptomic analyses
Source: Nat Commun. 2024 Apr 17;15:3306. doi: 10.1038/s41467-024-47484-1 (PMC11024210; doi:10.1038/s41467-024-47484-1)
Supplement: Supplementary file 3 — Description of Additional Supplementary Information [file 41467_2024_47484_MOESM3_ESM.pdf]

Description of Additional Supplementary Files:

Supplementary Dataset 1: Orthologs for zebrafish genes by integrating information from eggNOG, Alliance Genome (https://www.alliancegenome.org/), Ensembl and Zfin (https://zfin.org/).

Supplementary Dataset 2: Orthologs for mouse genes by integrating information from eggNOG, Alliance Genome (https://www.alliancegenome.org/) and Ensembl.
