# Supplementary material for: Reconstruction of macroglia and adult neurogenesis evolution through cross-species single-cell transcriptomic analyses
Source: Nat Commun. 2024 Apr 17;15:3306. doi: 10.1038/s41467-024-47484-1 (PMC11024210; doi:10.1038/s41467-024-47484-1)
Supplement: Supplementary file 6 — Reporting Summary [file 41467_2024_47484_MOESM6_ESM.docx]

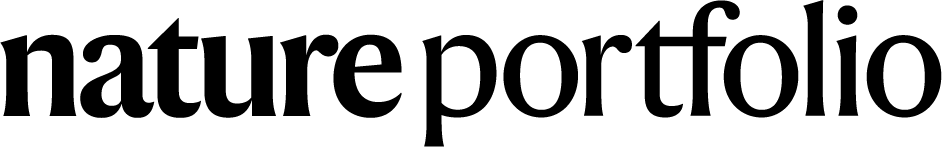
Corresponding author(s): Laure Bally-Cuif

Last updated by author(s): 2024/01/16

Reporting Summary

Nature Portfolio wishes to improve the reproducibility of the work that we publish. This form provides structure for consistency and transparency in reporting. For further information on Nature Portfolio policies, see our Editorial Policies and the Editorial Policy Checklist.

Please do not complete any field with "not applicable" or n/a. Refer to the help text for what text to use if an item is not relevant to your study. For final submission: please carefully check your responses for accuracy; you will not be able to make changes later.

## Statistics

For all statistical analyses, confirm that the following items are present in the figure legend, table legend, main text, or Methods section.

n/a


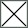


Confirmed


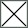
 The exact sample size (*n*) for each experimental group/condition, given as a discrete number and unit of measurement


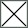
 A statement on whether measurements were taken from distinct samples or whether the same sample was measured repeatedly The statistical test(s) used AND whether they are one- or two-sided

*Only common tests should be described solely by name; describe more complex techniques in the Methods section.*


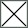
 A description of all covariates tested


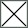
 A description of any assumptions or corrections, such as tests of normality and adjustment for multiple comparisons

A full description of the statistical parameters including central tendency (e.g. means) or other basic estimates (e.g. regression coefficient) AND variation (e.g. standard deviation) or associated estimates of uncertainty (e.g. confidence intervals)

For null hypothesis testing, the test statistic (e.g. *F*, *t*, *r*) with confidence intervals, effect sizes, degrees of freedom and *P* value noted

1

nature portfolio | reporting summary

*April 2023*

*Give P values as exact values whenever suitable.*

For Bayesian analysis, information on the choice of priors and Markov chain Monte Carlo settings

For hierarchical and complex designs, identification of the appropriate level for tests and full reporting of outcomes Estimates of effect sizes (e.g. Cohen's *d*, Pearson's *r*), indicating how they were calculated

*Our web collection on statistics for biologists contains articles on many of the points above.*

## Software and code

Policy information about availability of computer code Data collection

Data were either produced during the study or recovered from public databases without requiring any custom code.

Data analysis

All open access and custom codes used are described in the Materials and Methods and in the data.gouv repository (https:// entrepot.recherche.data.gouv.fr/privateurl.xhtml?token=bede1d62-f7cf-4e85-b6ce-05121176f108)

Software used for analysis included :

Cellranger 3.0.1 (used to process raw sequencing data and obtain count matrices) R 3.6.3

Seurat 2.3.4

scran 1.10.2

scater 1.10.1

M3Drop 1.8.1

DoubletFinder 2.0.0

DoubletDecon 0.0.0.9000

densityClust 0.3

dbscan 1.1-3

ADPclust 0.7

depmixS4 1.3-5

mclust 5.4.2

igraph 1.2.5

RANN 2.6.1

MAST 1.8.2

2

nature portfolio | reporting summary

*April 2023*

singleCellHaystack 0.2.0

GSEABase 1.44.0

liger 1.0

hypergate 0.8

pROC 1.13.0

ROCR 1.0-7

randomForest 4.6-14

SingleCellExperiment 1.4.1

scrattch.hicat 1.0.0

scrattch.vis 0.0.210

clusterExperiment 2.2.0

clusterProfiler 3.10.1

ClusterSignificance 1.10.0

MUDAN 0.1.0

destiny 2.12.0

diffusionMap 1.1-0.1

ica 1.0-2

irlba 2.3.3

Rtsne 0.15

uwot 0.1.11

ElPiGraph.R 1.0.0

DDRTree 0.1.5

dendextend 1.10.0

dynamicTreeCut 1.63-1

limma 3.38.3

MetaNeighbor 1.2.1

ggplot2 3.3.5

cowplot 0.9.4

patchwork 1.1.1

rgl 0.99.16

pheatmap 1.0.12

paleotree 3.4.5

RColorBrewer 1.1-2

viridis 0.5.1

scales 1.0.0

devtools 2.0.1

LoomExperiment 1.0.4

loomR 0.2.1.9000

rhdf5 2.26.2

Matrix 1.4-1

rmarkdown 1.11

Image analysis was conducted using Imaris 10.1.0

Lists of orthologs were obtained by pooling information from eggNOG, zfin, Ensembl, Alliance of Genome Ressources and flybase. Manually curated ortholog tables were produced for zebrafish and mouse and are available as supplementary data.

For manuscripts utilizing custom algorithms or software that are central to the research but not yet described in published literature, software must be made available to editors and reviewers. We strongly encourage code deposition in a community repository (e.g. GitHub). See the Nature Portfolio guidelines for submitting code & software for further information.

## Data

Policy information about availability of data

All manuscripts must include a data availability statement. This statement should provide the following information, where applicable:

- Accession codes, unique identifiers, or web links for publicly available datasets
- A description of any restrictions on data availability
- For clinical datasets or third party data, please ensure that the statement adheres to our policy

All data, code, and materials generated in this study are available at: https://entrepot.recherche.data.gouv.fr/privateurl.xhtml?token=bede1d62-f7cf-4e85-

b6ce-05121176f108. This site includes rds files for our dataset as well as most of the re-analyzed datasets unless. Re-analyzed datasets were not included only if 1) they are already readily available through the original publication, 2) our analyses agree with that of the original publication and 3) we did not conduct analyses requiring further partition of the original data. Although most of the analyses were conducted using Seurat v2.3.4 to store the data, we provide them in list format with accompanying custom plotting functions so that they can be downloaded and used with minimal coding experience and independently of the specific local R installation. We also provide html file to directly query the expression of genes relevant in our analyses, in particular the reconstruction of gene expression along the neurogenic cascade and the identification of cells expressing genes belonging to the astrocytic geneset, across the different datasets. Finally, the site includes a README file detailing the different datasets there, how to download them and how to extract raw or normalized data and/or plot genes of interest.

3

nature portfolio | reporting summary

*April 2023*

## Research involving human participants, their data, or biological material

Policy information about studies with human participants or human data. See also policy information about sex, gender (identity/presentation), and sexual orientation and race, ethnicity and racism.

NR (no human participants or data)

Reporting on sex and gender

Reporting on race, ethnicity, or other socially relevant groupings

NR (no human participants or data)

Population characteristics

NR (no human participants or data)

Recruitment

NR (no human participants or data)

Ethics oversight

NR (no human participants or data)

Note that full information on the approval of the study protocol must also be provided in the manuscript.

# Field-specific reporting

Please select the one below that is the best fit for your research. If you are not sure, read the appropriate sections before making your selection.


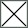
 Life sciences
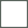
 Behavioural & social sciences
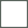
 Ecological, evolutionary & environmental sciences

For a reference copy of the document with all sections, see nature.com/documents/nr-reporting-summary-flat.pdf

# Life sciences study design

All studies must disclose on these points even when the disclosure is negative. Sample size

No precise sample sizes were calculated. The number of fish used for single-cell RNAseq took into account 1) our goal to be able to identify a cell population representing roughly 5% of the neural stem cell pool which based on rare cell clusters being readily identified in the literature and in our own previous analyses with a few dozen cells suggested that we needed at least 1000 neural stem cells, 2) 10X Chromium's capture efficiency and 3) the proportion of viable cells after telencephalon dissection and FACS determined by pilot experiments. Based on this, and in order to account for a probable lower cell capture efficiency than with murine or human cells we aimed at recovering roughly 10 000 cells per replicate, with 50% of these cells being sorted for high expression of GFP in the sox2:gfp line to enrich for neural stem and progenitor cells.

We recovered over 3000 neural stem cells with this collection strategy, enabling us to characterize their heterogeneity. On the other hand, our enrichment method means that we discarded many non-glial cells and that our dataset is less suited than others to fully explore neuronal diversity.

Data exclusions

Cells were excluded from further analysis based on both pre-specified criteria (number of genes < 250, percentage of a library made up of mitochondrial genes < 10…) and on dataset specific features (number of genes detected as a function of number of UMIs beyond three standard deviations from the mean of a fitted loess model). All datasets were used initially but one of the replicates was excluded from subclustering and comparative analyses. (We noticed lower numbers of detected genes per cell with a higher percentage of reads mapping to mitochondrial genes in cells from this replicate, which was consistent with a longer processing time during library preparation.)

Replication

3 replicates (2 for deep subclustering) were collected and yielded consistent results. The overall partition of the data was very similar between replicates when analyzed independently and also when they were pooled together despite independent parameter selection. Results were consistent with three different methods of highly variable genes selection and with mapping to two different transcriptome assembly (GRCz11 from Ensembl and the one developped by the Lawson lab)

Moreover we analyzed similar datasets (albeit with fewer cells, such as the one from the Kizil lab) generated from other labs which also yielded similar results. Finally we conducted extensive in situ validation experiments of our scRNAseq data.

Randomization

Fish were selected only on the basis of their age to focus on adult neurogenesis and on whether they expressed relevant transgenes. They were selected at random among suitable fish in our animal facility with no exclusion criteria based on gender. No other specific parameters were taken into account when selecting fish for experiments.

Blinding

For scRNA-seq no blinding was necessary as no comparisons between groups were performed. For timp4.3 smFISH quantification cells were first identified on the basis of clonal reporter expression or at random in Dm and timp4.3 levels of expression were later quantified without knowing which type of cell was being counted. Cell condition was then revealed after quantification. However all the steps were performed by the same person.

4

nature portfolio | reporting summary

*April 2023*

# Behavioural & social sciences study design

All studies must disclose on these points even when the disclosure is negative.

Study description

*Briefly describe the study type including whether data are quantitative, qualitative, or mixed-methods (e.g. qualitative cross-sectional, quantitative experimental, mixed-methods case study).*

Research sample

*State the research sample (e.g. Harvard university undergraduates, villagers in rural India) and provide relevant demographic information (e.g. age, sex) and indicate whether the sample is representative. Provide a rationale for the study sample chosen. For studies involving existing datasets, please describe the dataset and source.*

Sampling strategy

*Describe the sampling procedure (e.g. random, snowball, stratified, convenience). Describe the statistical methods that were used to predetermine sample size OR if no sample-size calculation was performed, describe how sample sizes were chosen and provide a rationale for why these sample sizes are sufficient. For qualitative data, please indicate whether data saturation was considered, and what criteria were used to decide that no further sampling was needed.*

Data collection

*Provide details about the data collection procedure, including the instruments or devices used to record the data (e.g. pen and paper, computer, eye tracker, video or audio equipment) whether anyone was present besides the participant(s) and the researcher, and whether the researcher was blind to experimental condition and/or the study hypothesis during data collection.*

Timing

*Indicate the start and stop dates of data collection. If there is a gap between collection periods, state the dates for each sample cohort.*

Data exclusions

*If no data were excluded from the analyses, state so OR if data were excluded, provide the exact number of exclusions and the rationale behind them, indicating whether exclusion criteria were pre-established.*

Non-participation

*State how many participants dropped out/declined participation and the reason(s) given OR provide response rate OR state that no participants dropped out/declined participation.*

Randomization

*If participants were not allocated into experimental groups, state so OR describe how participants were allocated to groups, and if allocation was not random, describe how covariates were controlled.*

# Ecological, evolutionary & environmental sciences study design

All studies must disclose on these points even when the disclosure is negative.

Study description

*Briefly describe the study. For quantitative data include treatment factors and interactions, design structure (e.g. factorial, nested, hierarchical), nature and number of experimental units and replicates.*

Research sample

*Describe the research sample (e.g. a group of tagged Passer domesticus, all Stenocereus thurberi within Organ Pipe Cactus National Monument), and provide a rationale for the sample choice. When relevant, describe the organism taxa, source, sex, age range and any manipulations. State what population the sample is meant to represent when applicable. For studies involving existing datasets, describe the data and its source.*

Sampling strategy

*Note the sampling procedure. Describe the statistical methods that were used to predetermine sample size OR if no sample-size calculation was performed, describe how sample sizes were chosen and provide a rationale for why these sample sizes are sufficient.*

Data collection

*Describe the data collection procedure, including who recorded the data and how.*

Timing and spatial scale

*Indicate the start and stop dates of data collection, noting the frequency and periodicity of sampling and providing a rationale for these choices. If there is a gap between collection periods, state the dates for each sample cohort. Specify the spatial scale from which the data are taken*

Data exclusions

*If no data were excluded from the analyses, state so OR if data were excluded, describe the exclusions and the rationale behind them, indicating whether exclusion criteria were pre-established.*

Reproducibility

*Describe the measures taken to verify the reproducibility of experimental findings. For each experiment, note whether any attempts to repeat the experiment failed OR state that all attempts to repeat the experiment were successful.*

Randomization

*Describe how samples/organisms/participants were allocated into groups. If allocation was not random, describe how covariates were controlled. If this is not relevant to your study, explain why.*

Blinding

*Describe the extent of blinding used during data acquisition and analysis. If blinding was not possible, describe why OR explain why blinding was not relevant to your study.*

Did the study involve field work? Yes No

5

nature portfolio | reporting summary

*April 2023*

## Field work, collection and transport

Field conditions

*Describe the study conditions for field work, providing relevant parameters (e.g. temperature, rainfall).*

Location

*State the location of the sampling or experiment, providing relevant parameters (e.g. latitude and longitude, elevation, water depth).*

Access & import/export

*Describe the efforts you have made to access habitats and to collect and import/export your samples in a responsible manner and in compliance with local, national and international laws, noting any permits that were obtained (give the name of the issuing authority, the date of issue, and any identifying information).*

Disturbance

*Describe any disturbance caused by the study and how it was minimized.*

# Reporting for specific materials, systems and methods

We require information from authors about some types of materials, experimental systems and methods used in many studies. Here, indicate whether each material, system or method listed is relevant to your study. If you are not sure if a list item applies to your research, read the appropriate section before selecting a response.

Materials & experimental systems Methods


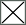

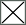

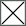

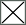

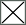


n/a Involved in the study Antibodies Eukaryotic cell lines

Palaeontology and archaeology Animals and other organisms Clinical data

Dual use research of concern

Plants


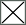

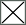


n/a Involved in the study

ChIP-seq

Flow cytometry

MRI-based neuroimaging

## Antibodies

Antibodies used

mCherry (Rabbit, Takara, #622496), ZO1 (Mouse monoclonal IgG1, Thermo Fisher Scientific, Cat#33-9100, RRID : 2533147)

Validation

These antibodies have been used in hundreds of publications. See for example Than-Trong et al. 2020 (ref24) and Mancini et al, Sci. Adv. 2023 doi: 10.1126/sciadv.adg7519

## Animals and other research organisms

Policy information about studies involving animals; ARRIVE guidelines recommended for reporting animal research, and Sex and Gender in Research

Laboratory animals

D.Rerio, AB strain, 3 months post-fertilization.

Wild animals

No wild animals were used in this study.

Reporting on sex

Mixed

Field-collected samples

No field-collected samples were used in this study.

Ethics oversight

All procedures relating to zebrafish (Danio rerio) care and treatment conformed to the directive 2010/63/EU of the European Parliament and of the council of the European Union. The animal study protocol was approved by the Ethics Committee n°39 of Institut Pasteur (authorization #36936, April 26th, 2022) and DDPP-2021-021 of the Direction Départementale de la Protection des Populations de Paris.

Note that full information on the approval of the study protocol must also be provided in the manuscript.

6

nature portfolio | reporting summary

*April 2023*

## Plants

Seed stocks

*Report on the source of all seed stocks or other plant material used. If applicable, state the seed stock centre and catalogue number. If plant specimens were collected from the field, describe the collection location, date and sampling procedures.*

*Describe the methods by which all novel plant genotypes were produced. This includes those generated by transgenic approaches, gene editing, chemical/radiation-based mutagenesis and hybridization. For transgenic lines, describe the transformation method, the number of independent lines analyzed and the generation upon which experiments were performed. For gene-edited lines, describe the editor used, the endogenous sequence targeted for editing, the targeting guide RNA sequence (if applicable) and how the editor was applied.*

*Describe any authentication procedures for each seed stock used or novel genotype generated. Describe any experiments used to*

*assess the effect of a mutation and, where applicable, how potential secondary effects (e.g. second site T-DNA insertions, mosiacism, off-target gene editing) were examined.*

Novel plant genotypes

Authentication

## Flow Cytometry

### Plots

Confirm that:


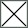
 The axis labels state the marker and fluorochrome used (e.g. CD4-FITC).


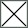
 The axis scales are clearly visible. Include numbers along axes only for bottom left plot of group (a 'group' is an analysis of identical markers).
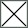
 All plots are contour plots with outliers or pseudocolor plots.

A numerical value for number of cells or percentage (with statistics) is provided.

### Methodology

Sample preparation

Cell sorting was conducted three days in a row to collect replicates, using twenty 3 months old adults from the Tg(sox2::GFP) line on each day. Brains were dissected in Ringer's solution. The telencephalon was separated from the midbrain and the olfactory bulbs were removed. The two hemispheres were separated and cut along the boundary between pallium and subpallium to enrich for pallial cells. Cell dissociation was carried out according to Manoli, M. & Driever, W. Fluorescence- Activated Cell Sorting (FACS) of Fluorescently Tagged Cells from Zebrafish Larvae for RNA Isolation. Cold Spring Harb Protoc 2012, pdb.prot069633 (2012).

Instrument

FACSAria III

Software

BD FACSDiva 8.0.1

Cell population abundance

Details on cell abundance can be found in the supplementary data for each replicate. A preliminary test was conducted to confirm that a second run on the same cytometer from freshly sorted cells yielded similar results and that assessment of GFP

+ cells and viability was comparable when using a Malassez slide.

Gating strategy

Details about gating strategy for each replicate can be found in the supplementary data. Cutoffs for viability and GFP expression were set on a small subsample priori to conducting the actual experiments and then applied to cells of interest.

Tick this box to confirm that a figure exemplifying the gating strategy is provided in the Supplementary Information.


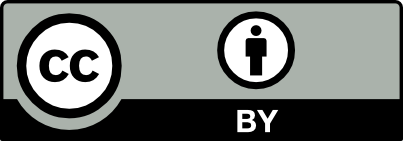
This checklist template is licensed under a Creative Commons Attribution 4.0 International License, which permits use, sharing, adaptation, distribution and reproduction in any medium or format, as long as you give appropriate credit to the original author(s) and the source, provide a link to the Creative Commons license, and indicate if changes were made. The images or other third party material in this article are included in the article's Creative Commons license, unless indicated otherwise in a credit line to the material. If material is not included in the article's Creative Commons license and your intended use is not permitted by statutory regulation or exceeds the permitted use, you will need to obtain permission directly from the copyright holder. To view a copy of this license, visit <http://creativecommons.org/licenses/by/4.0/>
